# Urine Metabolomics Exposes Anomalous Recovery after Maximal Exertion in Female ME/CFS Patients

**DOI:** 10.3390/ijms24043685

**Published:** 2023-02-12

**Authors:** Katherine A. Glass, Arnaud Germain, Yuhsin V. Huang, Maureen R. Hanson

**Affiliations:** Department of Molecular Biology and Genetics, Cornell University, Ithaca, NY 14853, USA

**Keywords:** ME/CFS, metabolomics, urine, exercise

## Abstract

Myalgic encephalomyelitis/chronic fatigue syndrome (ME/CFS) is a debilitating disease with unknown etiology or effective treatments. Post-exertional malaise (PEM) is a key symptom that distinguishes ME/CFS patients. Investigating changes in the urine metabolome between ME/CFS patients and healthy subjects following exertion may help us understand PEM. The aim of this pilot study was to comprehensively characterize the urine metabolomes of eight female healthy sedentary control subjects and ten female ME/CFS patients in response to a maximal cardiopulmonary exercise test (CPET). Each subject provided urine samples at baseline and 24 h post-exercise. A total of 1403 metabolites were detected via LC-MS/MS by Metabolon^®^ including amino acids, carbohydrates, lipids, nucleotides, cofactors and vitamins, xenobiotics, and unknown compounds. Using a linear mixed effects model, pathway enrichment analysis, topology analysis, and correlations between urine and plasma metabolite levels, significant differences were discovered between controls and ME/CFS patients in many lipid (steroids, acyl carnitines and acyl glycines) and amino acid subpathways (cysteine, methionine, SAM, and taurine; leucine, isoleucine, and valine; polyamine; tryptophan; and urea cycle, arginine and proline). Our most unanticipated discovery is the lack of changes in the urine metabolome of ME/CFS patients during recovery while significant changes are induced in controls after CPET, potentially demonstrating the lack of adaptation to a severe stress in ME/CFS patients.

## 1. Introduction

Myalgic encephalomyelitis/chronic fatigue syndrome (ME/CFS) is a debilitating disease affecting an estimated 1.5–3 million adults and children in the United States alone [1,2]. The majority of ME/CFS patients are unable to work due to their illness, leading to an estimated minimum economic impact of 35–51 billion USD per year in medical costs and lost productivity combined [1]. Symptoms of this acquired, systemic disease include a new onset of persistent physical and mental fatigue severe enough to prevent normal activities, unrefreshing sleep, pain, cognitive impairment, orthostatic intolerance, immune manifestations such as recurrent flu-like symptoms and sore throat, and neuroendocrine manifestations such as intolerance to heat and cold [3].

In addition, the hallmark symptom of ME/CFS is post-exertional malaise (PEM), which is a worsening of symptoms after any type of exertion, including both physical and cognitive exertion, beginning from immediately following the exertion to more than 24 h later [4]. PEM may last hours to months, and the duration varies extensively even for individual patients [4,5]. Therefore, unlike most chronic illnesses, in which exercise is beneficial, people with ME/CFS are exercise intolerant. This exercise intolerance can be clinically assessed using a two-day cardiopulmonary exercise test (CPET).

In the two-day CPET protocol, the first CPET is used to measure “baseline functional capacity” while inducing PEM [6]. The second CPET, 24 h later, measures the impaired performance at a time when most patients will already be experiencing PEM symptoms from the first CPET. Subjects with other various illnesses are able to perform similarly on a CPET two days in a row, whereas ME/CFS patients are unable to perform as well the second day [7,8]. This reduced performance ability can be documented with objective measures including reduced maximal oxygen consumption and peak workload greater than the typical variability of repeated testing, despite subjects maintaining a respiratory exchange ratio (RER) above 1.1, which corresponds to maximum effort [6].

Although there is a growing body of knowledge describing the pathophysiology of ME/CFS, the etiology of the disease remains unknown and there are currently no diagnostic laboratory tests nor FDA-approved treatments. Despite the lack of diagnostic biomarkers, there are many documented molecular pathophysiological changes occurring in ME/CFS, including in metabolomics [9,10].

The plasma metabolome of ME/CFS patients has received a substantial amount of attention for over a decade, although often on limited cohort sizes, with an increasing number of metabolites measured (from just over 20 to about 1200 metabolites more recently) [11,12,13,14,15,16,17,18,19,20,21,22]. On the contrary, previous studies of urine metabolomics in ME/CFS patients are very limited. The few published studies have measured only 28–42 metabolites, which have primarily been amino acids [19,20,23,24]. While most findings have not been consistent between studies, two studies did find phenylalanine at lower concentrations in ME/CFS patients than healthy controls [23,24]. Although one study by McGregor et al. examined urine metabolites in the context of self-reported PEM [19], and our group recently published a thorough investigation of the plasma metabolome (1157 metabolites) before and after exercise [25], no studies of ME/CFS patients have measured metabolites in urine after a deliberate exercise challenge.

Measuring compounds in urine is advantageous due to non-invasive and easy sample collection which makes it ideal for diagnostics. Additionally, altered excretion of metabolites in ME/CFS patients after an exercise challenge may yield insights into the pathophysiology of PEM that complement the changes documented in the plasma metabolome. The aim of this pilot study was to comprehensively investigate changes in the urine metabolomes of eight female healthy sedentary control subjects and ten female ME/CFS patients in response to a maximal cardiopulmonary exercise test (CPET). This study represents an approximate 30-fold increase in the number of metabolites measured in the urine of ME/CFS patients, from less than 50 in previous studies to 1403 in the current study.

Our extensive analysis reveals numerous and significant differences in the urine metabolomes of ME/CFS and control groups in response to exercise, despite the small number of subjects studied. Such changes are predominantly present in the lipid and amino acid metabolic superpathways. We found a large number of metabolites with increased levels in the urine of controls 24 h post-exercise. The post-exercise increase in urinary excretion did not occur in the ME/CFS patients, which is evidence of a metabolic dysregulation during exercise recovery.

## 2. Results

### 2.1. Study Design and Subject Characteristics

**Figure 1:**
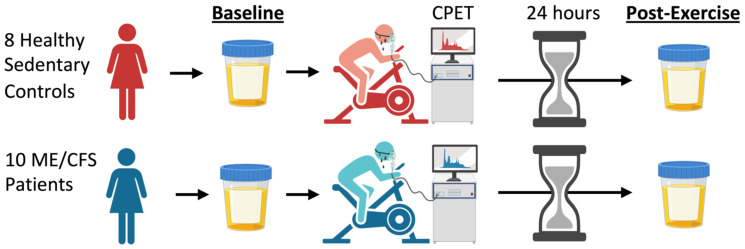
**Study schematic.** Eight sedentary healthy controls and ten ME/CFS patients were included in this study. ME/CFS patients were diagnosed with the Canadian Consensus Criteria. All subjects provided a baseline urine sample in the morning prior to exercise testing and 24 h later. Schematic created with Biorender.com.

Eight female healthy sedentary control subjects and ten female ME/CFS patients provided a baseline urine sample in the morning prior to exercise testing (Figure 1). All subjects performed the CPET on a stationary bicycle and were monitored to ensure that they used maximal effort (RER > 1.1). A post-exercise urine sample was collected from all subjects 24 h later. Metabolites were measured in all urine samples by Metabolon^®^ using their Precision Metabolomics™ LC-MS/MS global metabolomics platform.

Table 1 shows the demographic information for all of the subjects. The subjects were highly age-matched with median ages of 52.5 and 51.5 years in the control and ME/CFS groups, respectively. The ME/CFS group had significantly lower BMI than the control group. Only one participant identified as Hispanic (an ME/CFS patient) and most subjects identified as white, except for one Asian ME/CFS subject.

As expected, the ME/CFS patients scored significantly lower on multiple measures of physical function, including the Bell disability scale and the SF-36 physical component. On the Bell scale, the median for the controls was 90, which corresponds to “No symptoms at rest; mild symptoms with activity; normal overall activity level; able to work full-time without difficulty” [26]. The median for the ME/CFS patients was 30, which corresponds to “Moderate to severe symptoms at rest. Severe symptoms with any exercise; overall activity level reduced to 50% of expected. Usually confined to house. Unable to perform any strenuous tasks. Able to perform desk work 2–3 h a day, but requires rest periods” [26]. The ME/CFS patients had a wide range of disease duration of 2–27 years (median 7.5 years).

### 2.2. Many Differences in the Urine Metabolomes of ME/CFS Patients and Controls Emerge through Analysis of Changes between Pre- and Post-Exercise Samples

Metabolon^®^ detected a total of 1403 metabolites in these samples using their Precision Metabolomics™ LC-MS/MS global metabolomics platform. Out of the 1403 metabolites measured, 886 are known metabolites, 64 are partially characterized molecules, and 453 are unknown compounds (Supplementary File S1—Raw Data). All data were osmolality normalized to account for differences in overall concentration of each urine sample. Osmolality data for each experimental group are shown in Supplementary Figure S1 and was not significantly different between ME/CFS patients and controls at either time point. There was a trend toward increased osmolality in the controls 24 h post-exercise (*p* < 0.1, linear mixed effects model, followed by pairwise comparisons with Tukey’s posthoc test). By normalizing to the osmolality, we ensured that any differences detected in metabolite levels both between ME/CFS and control groups and from baseline to post-exercise within cohorts are not simply a reflection of changes in osmolality.

Missing values were imputed with the minimum as recommended by Metabolon^®^, except in the case of drugs and tobacco, where the missing values were imputed with 0. The data for each metabolite were median centered to 1, and all data were log10 transformed using MetaboAnalystR (available at www.metaboanalyst.ca, accessed on 14 December 2022). Filtering was applied to eliminate compounds with a large amount of missing data from the analysis according to the modified 80% rule: a metabolite is included if it is detected in at least 80% of the samples in either the ME/CFS patients, the controls, or both groups [27]. In total, 1154 metabolites met the criteria and were included in the analysis.

A linear mixed effects model (LMM) for each metabolite was utilized to determine the differences between ME/CFS and control groups at each time point (baseline or post-exercise), the change over time within the ME/CFS and control groups, and which metabolites were changing differently after exercise in the ME/CFS and control groups. The model formula is as follows:

Metabolite ~ Disease status ∗ Time Point + Age + BMI + (1|Subject)

The *p*-values for each metabolite were adjusted for multiple comparisons using the Benjamini–Hochberg (BH) procedure with a significance threshold of *q* < 0.1 (Supplementary File S2—Linear Mixed Model Results) [28]. Because we adjusted for the confounders age and BMI, the significant differences we detected with this model are not due to the difference in BMI in the two groups.

**Figure 2:**
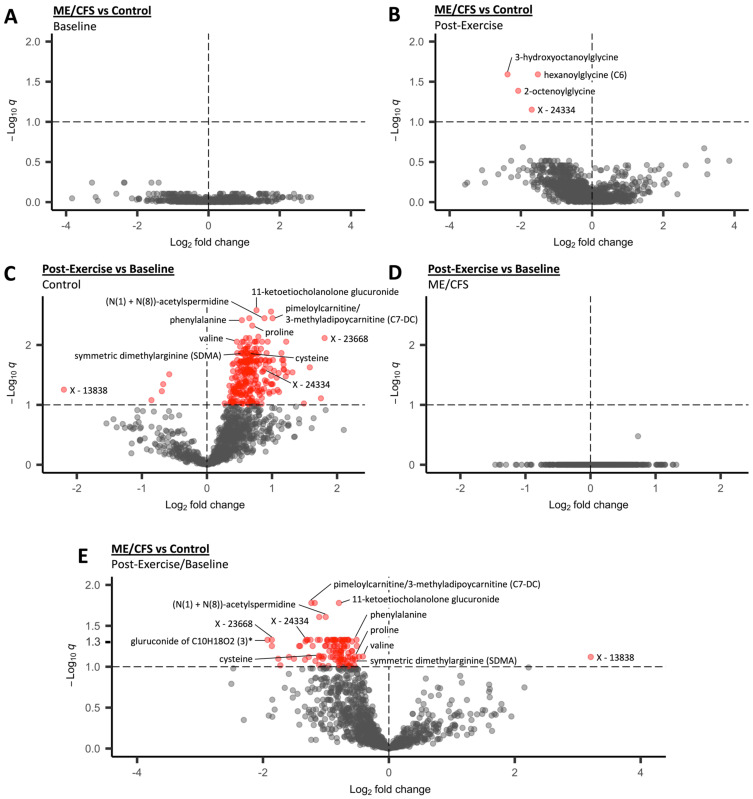
**Linear mixed modeling results (A–E).** The *p*-values from within and between group contrasts were adjusted for multiple comparisons using the BH procedure. Each dot represents one of 1154 metabolites that passed filtering. The y axis shows the negative log of the *q* value, so a higher number represents compounds that are more statistically significant. The dashed horizontal line shows the significance threshold *q* < 0.1 and the red dots are the compounds that are significant at that threshold. The gray dots are compounds that are not significant. The x axis shows the log2 fold change for each comparison.

No significant differences were detected between controls and ME/CFS patients at baseline (Figure 2A). At 24 h post-exercise, four compounds were significantly different, all of which were at lower concentrations in the ME/CFS patients compared to the controls (Figure 2B). The four compounds included three acyl glycines and one unknown compound.

The control group exhibited large-scale changes in the urine metabolome when comparing the baseline and post-exercise urine samples, with 255 compounds significantly altered at the *q* < 0.1 threshold (Figure 2C). All except five compounds showed increased concentrations after exercise. This is in stark contrast to the ME/CFS group, in which we did not detect any compounds with significant changes in concentration due to exercise (Figure 2D).

A significant interaction between disease status (ME/CFS vs. control) and time point (baseline vs. post-exercise) in the linear mixed effects model shows which metabolites are changing differently in the ME/CFS and control groups during exercise recovery (i.e., over time). Figure 2E displays 110 significantly different metabolites at the *q* < 0.1 threshold (red dots), and 35 metabolites are also below *q* < 0.05 (1.3 on the -Log_10_*q* y-axis of Figure 2E). In this volcano plot, the log2 fold change is a ratio of ratios; the ratio of the mean post-exercise/baseline ratios in the ME/CFS patients to the mean post-exercise/baseline ratios in the controls. The post-exercise/baseline ratio for each subject shows whether the metabolite is increased or decreased in urine in each subject during exercise recovery. The mean normalized concentrations for the controls and ME/CFS patients at both time points for the 56 known compounds included in these 110 compounds show that for most compounds, there is a post-exercise increase in the controls that is not seen in the ME/CFS patients (Supplementary Figure S2). Therefore, the compounds which are changing differently during recovery in the control and ME/CFS groups are predominantly increased post-exercise in the control group and not significantly altered in the ME/CFS group, leading to a negative log2 fold change for the ME/CFS vs. control post-exercise/baseline ratios. In total, 102 of the 110 compounds that are changing differently over time in patients vs. controls are also significantly increased in the controls post-exercise.

The fact that there are metabolite changes in the controls and not in the patients is not due to increased variation in metabolite levels in the ME/CFS patients compared to the controls. There is no trend toward higher standard deviation in the ME/CFS group when comparing the standard deviations for ME/CFS to controls for each compound (Supplementary Figure S3). Additionally, these changes are detected despite normalizing to urine osmolality, which is trending toward an increase in the controls from baseline to post-exercise (Supplementary Figure S1). In order to increase significantly after normalization, levels of a metabolite have to increase even higher than any increase in overall urine concentration.

Together, these results demonstrate that exercise induces a significant increase in many metabolites in urine 24 h post-exercise in healthy sedentary controls, and the lack of these changes in the ME/CFS patients is a key component of their disease state and could be related to exercise intolerance.

### 2.3. Two Approaches to Enrichment Analysis Reveal Metabolic Subpathways with the Most Significant Changes

We then performed enrichment analysis using Metabolon^®^’s pathway annotations and ChemRICH (in R) [29,30]. This analysis does not rely on any background or reference metabolome and contains non-overlapping pathway assignments for each metabolite. Unlike the standard ChemRICH analysis which assigns metabolites to clusters by chemical similarity, this analysis used non-overlapping pathway assignments by Metabolon^®^. By looking at the pathways as opposed to single metabolites, we can identify metabolite sets which are significantly altered as a group, whereas individual metabolites in that set may not have achieved significance in the LMM on their own. Additionally, we can holistically describe the changes in the urine metabolome occurring post-exercise and how they are significantly altered in the ME/CFS patients. The input for this analysis is the fold change of the mean normalized concentration for each comparison and the *p*-values from the LMM contrasts for the 734 known compounds out of 1154 total analyzed (assigned to 84 subpathways out of 92 subpathways for all detected metabolites). Enrichment is assessed using the Kolmogorov–Smirnov test, with BH FDR correction (significance threshold *q* < 0.05).

**Figure 3:**
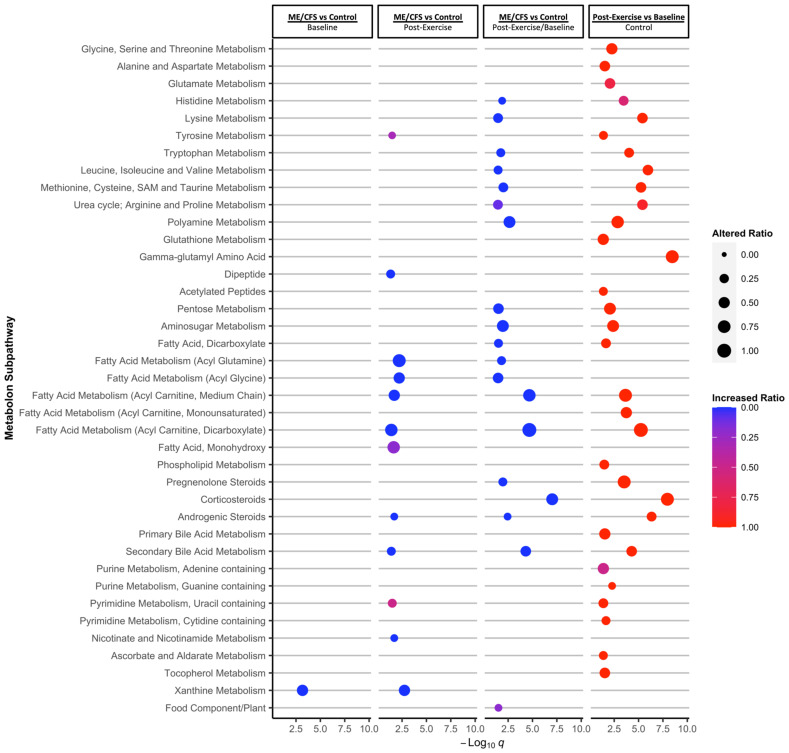
**Metabolon subpathway enrichment analysis.** Shown are Metabolon^®^ subpathways that were significantly altered for each comparison (Kolmogorov–Smirnov test, *q* < 0.05, BH FDR correction). The clusters are ordered by chemical similarity according to Metabolon^®^. Bubble size shows the ratio of significantly altered metabolites to total metabolites in that cluster (*p* < 0.05). The color gradient shows the increased ratio, where blue indicates all of the altered metabolites were decreased, and red indicates all altered compounds were increased. The post-exercise vs. baseline comparison for the ME/CFS patients was also evaluated but no significantly altered pathways were identified. All 734 identified metabolites assigned to 84 subpathways were included. Analysis was performed using ChemRICH in R.

Figure 3 shows the significantly altered subpathways for the same comparisons as in Figure 2: ME/CFS vs. controls at baseline, post-exercise, and for the post-exercise/baseline ratios, and the change post-exercise in the control group alone. We also analyzed the change post-exercise in the ME/CFS patients, but there were no significantly altered pathways.

In this figure, the subpathways are organized by Metabolon^®^ by chemical similarity and then superpathways are organized alphabetically, with amino acids first, followed by carbohydrates, lipids, nucleotides, vitamins and cofactors, and finally xenobiotics. An altered ratio of 1 means every compound we measured in a pathway was significantly altered (*p* < 0.05 in the LMM). Red bubbles indicate that all significantly altered compounds increased in that comparison, and blue bubbles indicate that all significantly altered compounds decreased in that comparison. We also performed ChemRICH analysis using the Medical Subject Headings (MeSH) ontology and Simplified Molecular Input Line Entry System (SMILES) codes to assign compounds to clusters based on chemical similarity (Supplementary Figure S4). Only 516 compounds could be matched to SMILES codes for this analysis. We chose a significance threshold of *q* < 0.15 for the MeSH ontology enrichment, because we did not want to exclude potentially interesting findings for this pilot study, and at *q* < 0.15 all clusters originally had *p* < 0.025. For the Metabolon^®^ subpathway enrichment, only at *q* < 0.05 were all significant clusters also at *p* < 0.05.

At baseline, only xanthine metabolism and the xanthines’ chemical cluster were significantly different between ME/CFS patients and controls, with lower concentrations of all compounds found in the urine of ME/CFS patients (Figure 3, *q* < 0.05 and Supplementary Figure S4, *q* < 0.15). Xanthines include caffeine and theophylline, so this chemical cluster is affected by diet.

Twelve Metabolon^®^ subpathways were significantly altered post-exercise in the ME/CFS patients vs. controls, again with predominantly lower metabolite concentrations in the ME/CFS patients (Figure 3). Five of these subpathways belong to the lipid super-pathway and are involved in fatty acid metabolism (including acyl glutamine, acyl glycine, and acyl carnitine). Within the amino acid metabolism superpathway, tyrosine metabolism was significantly different 24 h post-exercise and within nucleotide metabolism, uracil containing pyrimidine metabolism was significantly altered. 24 h post-exercise, only three chemical clusters were significantly altered in the ME/CFS patients using the MeSH ontology, including fatty acids (10:1), xanthines, and sugar acids (Supplementary Figure S4).

When comparing the exercise recovery (the change over time) in ME/CFS patients vs. controls using the post-exercise/baseline ratios, even more pathways are found to be significantly different, namely seven amino acid subpathways, seven lipid subpathways, and two carbohydrate subpathways. For this comparison, there are 13 chemical clusters which are significantly different between patients and controls (Supplementary Figure S4).

The highest number of significantly altered Metabolon^®^ subpathways and chemical clusters was found in the control group when comparing the post-exercise and baseline time points (Figure 3 and Figure S4), with the large majority of compounds increased post-exercise (red color). Only the adenine containing purine metabolism subpathway, the histidine metabolism subpathway, and the methylhistidines chemical cluster had a similar amount of increased and decreased compounds.

Most of the Metabolon^®^ subpathways and chemical clusters increased in the controls after exercise are the same ones that were significantly altered in the ME/CFS vs. controls when comparing the post-exercise/baseline ratio, showing that they were changing differently during exercise recovery in the ME/CFS patients vs. healthy sedentary controls. For both of these comparisons, the subpathway with the lowest *q*-value for subpathway enrichment was corticosteroids. Overall, 8/13 compounds were significantly altered in the ME/CFS patients vs. controls (and all had lower post-exercise/baseline ratios in patients), and 11/13 compounds significantly increased in controls after exercise (*p* < 0.05 in the LMM).

Four subpathways, all belonging to the lipid superpathway, had significantly altered concentration in urine in all three comparisons: ME/CFS vs. control at the post-exercise time point, ME/CFS vs. control post-exercise/baseline ratios, and post-exercise vs. baseline in the control group. These subpathways include two acyl carnitine fatty acid metabolism subpathways (medium chain and dicarboxylate), androgenic steroids, and secondary bile acid metabolism.

There are also several amino acid subpathways in which most altered compounds are significantly increased in the urine in controls post-exercise, and decreased when comparing the ME/CFS to control post-exercise/baseline ratios. Of these subpathways, the altered ratio is the highest for polyamine metabolism (and it has the lowest *q* value for the ME/CFS vs. controls): 6/9 metabolites are significantly increased in controls, and 5/9 are significantly decreased in the ME/CFS vs. controls post-exercise/baseline ratio. These compounds include acisoga, (N(1)+N(8))-acetylspermidine, diacetylspermidine, N1,N12-diacetylspermine, and N-acetyl-isoputreanine (for this subpathway, all compounds also have *q* < 0.1 in the LMM).

The arginine and proline metabolism subpathway of the urea cycle in the amino acid superpathway is the only one with a metabolite that is increased in ME/CFS vs. controls for the post-exercise/baseline ratios (besides food component/plant), with 8/10 metabolites significantly altered and methylurea being the only increased metabolite (*p* < 0.05 in LMM). Methylurea is also significantly decreasing in the controls over time (*p* < 0.05 in LMM), and is the only one of 11 altered compounds in this subpathway that is not increasing. However, the changes in methylurea were not significant when considering the univariate LMM analysis with the *q* < 0.1 threshold. Nevertheless, four compounds in this subpathway do have *q* < 0.1 in the LMM for the ME/CFS vs. controls post-exercise/baseline comparison: carboxy-methyl-arginine, proline, symmetric dimethylarginine, and asymmetric dimethylarginine.

The leucine, isoleucine, and valine metabolism subpathway has the lowest *q*-value in the controls of the subpathways in the amino acid superpathway. This subpathway has 36 compounds, with six changing differently over time in ME/CFS vs. controls: 3-methylglutarylcarnitine, tiglylcarnitine(C5), valine, beta-hydroxyisovalerate, beta-hydroxyisovaleroylcarnitine, and methylsuccinoylcarnitine. Sixteen out of thirty-six compounds are significantly increased in the controls, including all six changing differently over time in the ME/CFS patients vs. controls.

### 2.4. A Pathway Topology Analysis Highlights Altered Carbohydrates and Amino Acid Metabolism as a Result of Exercise

Differences between ME/CFS and control subjects at the metabolic pathway level were assessed using the pathway analysis module within the Metaboanalyst 5.0 webtool (www.metaboanalyst.ca, accessed on 14 December 2022), which combines quantitative enrichment analysis and pathway topology analysis. In total, 453 out of 734 known compounds of the 1154 compounds analyzed were included in this analysis, due to limitations in Human Metabolome Database (HMDB) ID matching. This analysis was carried out twice for each comparison, with the Kyoto encyclopedia of genes and genomes (KEGG) and then the small molecule pathway database (SMPDB) human reference metabolomes to define pathways. The quantitative enrichment analysis uses the global test to compare the two groups, which employs a logistic regression method to test whether the metabolites in the pathway help to improve classification of the samples as ME/CFS or control, with the null hypothesis that no metabolite in the pathway has a different concentration in either group [31,32]. The *p*-values from this test are adjusted for multiple comparisons using the BH procedure, with *q* < 0.2 as the significance threshold. This threshold was chosen to allow review of potentially interesting findings considering the small sample size, while ensuring that all significant pathways still have *p* < 0.05. This analysis is different from the ChemRICH enrichment analysis above in that it allows overlap, so several pathways may have the same key compounds. When evaluating the key compounds, we also report their significance as individual compounds in the LMM, where we employed a more stringent cutoff (*q* < 0.1). The outcome of the topology analysis is an impact score ranging from 0 to 1. This score is derived from metabolite node importance values calculated using the relative betweenness centrality measure, which are then normalized so that the maximum importance of each pathway is 1. The impact score is the sum of the importance measures for each matching metabolite node in a pathway.

**Figure 4:**
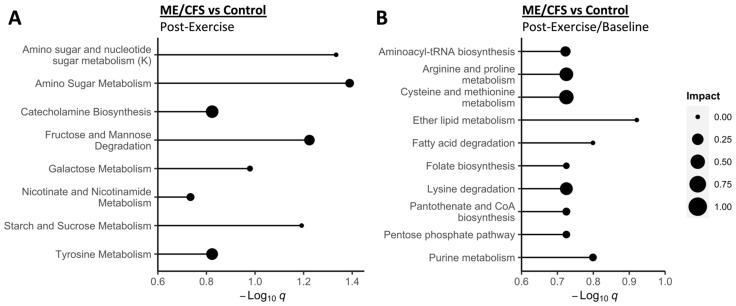
**Pathway topology and enrichment analysis (A,B).** Significantly different pathways for each comparison are listed in alphabetical order. The x axis shows the negative log of the *q*-values from BH FDR correction. All pathways shown have *q* < 0.2 (global test). The size of the bubble shows the impact score, which is a normalized measure of the importance of the altered metabolites in each pathway. ME/CFS and control groups were also compared at baseline but there were no significantly altered pathways after FDR correction. A total of 453 compounds were included in this analysis performed with Metaboanalyst 5.0. (**A**) For the post-exercise time point, all significant pathways were from the SMPDB reference metabolome except the one with (K) which was from KEGG. (**B**) For the post-exercise/baseline comparison, all significant pathways were from the KEGG reference metabolome.

Again, there were no significant differences between the urine metabolites at baseline in the ME/CFS and control groups. However, comparing ME/CFS patients and controls at the post-exercise time point and the change over time using the within subject post-exercise/baseline fold change, both had several significantly altered metabolic pathways (Figure 4).

At 24 h post-exercise, seven pathways in the SMPDB and one pathway in KEGG were significantly altered in the ME/CFS patients vs. controls (Figure 4A, *q* < 0.2). Five of the eight significantly altered pathways are involved in sugar metabolism, which is related to energy production. Fructose is the key compound in all of the sugar metabolism-related pathways, with *p* < 0.01 in the LMM when comparing the ME/CFS patients and controls post-exercise; however, fructose is not significant in the univariate analysis after multiple comparisons correction. The pathways with the highest impact scores are catecholamine biosynthesis followed by tyrosine metabolism. Both pathways have dehydroascorbate (*p* < 0.01) as a key compound, but again, this compound is not significant in the LMM after FDR correction. For tyrosine metabolism, ascorbate (vitamin C) (*p* < 0.01) is an additional key compound but is also not significant after FDR correction.

Ten pathways, all in the KEGG database, had significantly different post-exercise/baseline ratios in the ME/CFS patients vs. controls, indicating they are changing differently over time (Figure 4B, *q* < 0.2). The four pathways with the highest impact are all involved in amino acid metabolism: arginine and proline metabolism, cysteine and methionine metabolism, lysine degradation, and aminoacyl-tRNA biosynthesis. All of the proteinogenic amino acids are involved in the aminoacyl-tRNA biosynthesis pathway, so it makes sense that the pathway is significantly affected since several of these amino acids are significantly different in this comparison (see Supplementary Figure S2). For the KEGG arginine and proline metabolism pathway, proline is the only compound that is also significant in the LMM (*q* < 0.1) and also has one of the highest importance scores.

Cysteine is changing differently after exercise in the ME/CFS patients vs. controls in the LMM model (*q* < 0.1) and is the key compound in the KEGG pathways cysteine and methionine metabolism and pantothenate and Coenzyme A (CoA) biosynthesis. CoA is a ubiquitous cofactor that is required for fatty acid metabolism and the tricarboxylic acid (TCA) cycle. Its biosynthesis requires cysteine, which is a unique amino acid as it is the only one that contains a thiol group. Valine is also involved in the significantly altered pantothenate and Coenzyme A (CoA) biosynthesis pathway and has *q* < 0.1 in the LMM.

The most highly significant pathway for this comparison was ether lipid metabolism, but it had a low impact score. Although only two metabolites that we detected were matched to this pathway, they are both significant in the LMM with *q* < 0.1: glycerophosphoethanolamine and glycerophosphorylcholine (GPC) (both categorized as phospholipid metabolism by Metabolon^®^, see Supplementary Figure S2). Purine metabolism was also highly significant, with adenosine 3′,5′-cyclic monophosphate (cAMP) as the key compound (*q* < 0.1 in LMM).

### 2.5. Acyl Glycines Have Lower Concentrations in the Urine of ME/CFS Patients Compared to Controls 24 Hours Post-Exercise

**Figure 5:**
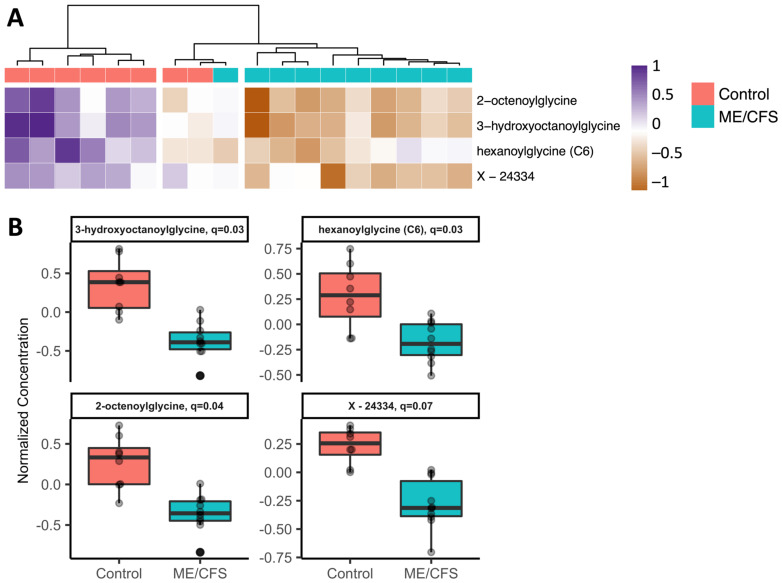
**The four metabolites found to be significantly different between controls and ME/CFS patients post-exercise.** (**A**) Heatmap showing hierarchical clustering of subjects for these 4 compounds. Colored rectangles show the normalized concentration of each metabolite for each subject 24 h post-exercise. (**B**) Boxplots showing the same data with the *q*-values for each compound.

The four metabolites found at significantly lower concentrations in ME/CFS vs. control subjects at the post-exercise time point in the univariate LMM analyses (*q* < 0.1) were 3-hydroxyoctanoylglycine, hexanoylglycine (C6), 2-octenoylglycine, and unknown X–24334. The three known compounds are all in the Metabolon^®^ subpathway acyl glycine fatty acid metabolism. The heatmap in Figure 5A shows the osmolality-normalized concentration post-exercise for these metabolites for every subject. Using agglomerative hierarchical clustering, the subjects clustered into three groups: (1) six control subjects, (2) two control subjects and one ME/CFS subject, and (3) the remaining nine ME/CFS subjects. The cluster of control subjects predominantly shows higher concentrations for all four metabolites while the cluster of ME/CFS subjects shows lower concentrations for all four metabolites. The small cluster with subjects from both groups shows intermediate values. The boxplots in Figure 5B demonstrate the minimal amount of overlap between the ME/CFS and control groups for these metabolites. Out of those four metabolites, only X-24334 is also changing significantly differently over time between controls and ME/CFS patients in the LMM and is increased after exercise in the control group.

### 2.6. Metabolites That Are Changing Differently during Exercise Recovery in ME/CFS Patients vs. Controls Are Predominantly Amino Acids and Lipids

**Figure 6:**
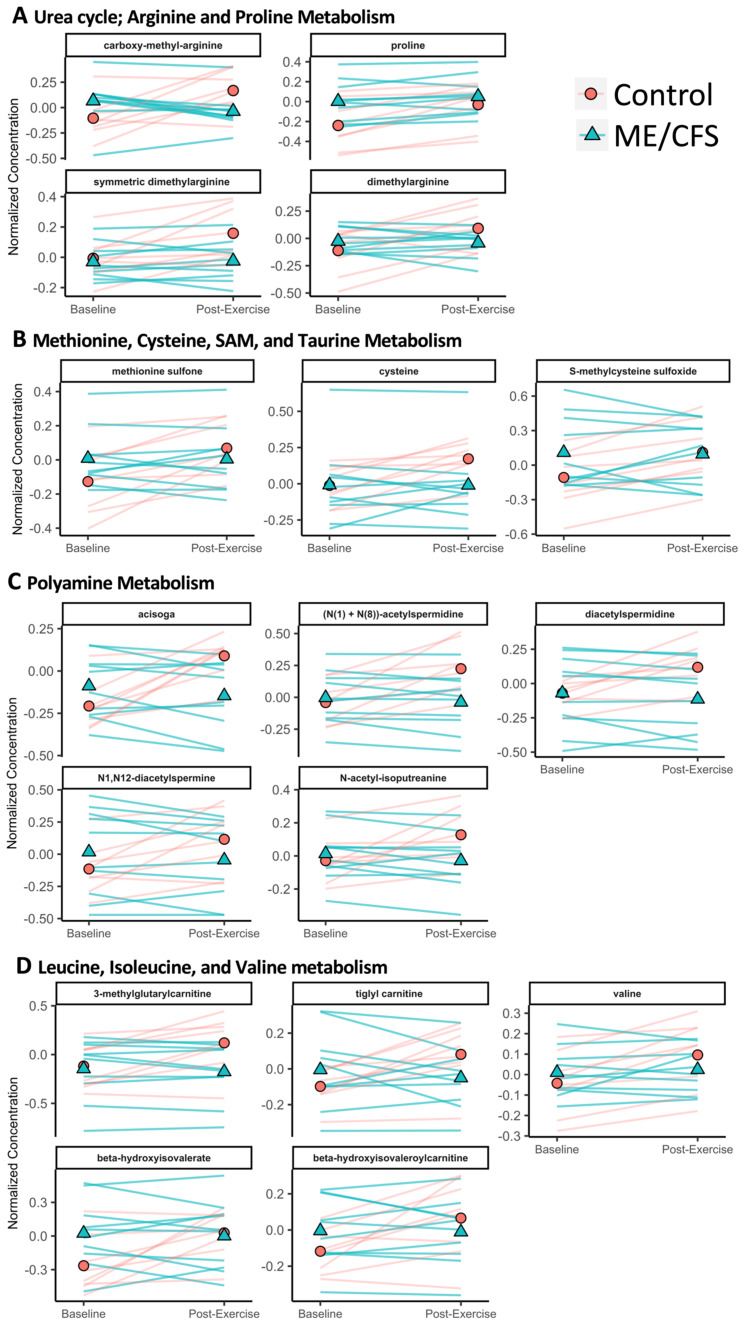
**Select amino acid compounds changing differently during exercise recovery in ME/CFS patients vs. controls.** (**A**–**D**) Shown are all data points for ME/CFS and healthy controls for both time points, baseline and post-exercise. The red circle and the blue triangle represent the mean at each time point of the healthy controls and the ME/CFS patients, respectively. Each line is one subject. All compounds shown have *q* < 0.1 in the interaction term of the LMM, and are also significantly increasing in the controls from baseline to post-exercise (*q* < 0.1). The compounds within each section are in order of lowest to highest *q*-value. (**A**) Urea cycle; arginine and proline metabolism, amino acid. (**B**) Methionine cysteine, SAM, and taurine metabolism. (**C**) Polyamine metabolism. (**D**) Leucine, isoleucine, and valine metabolism.

**Figure 7:**
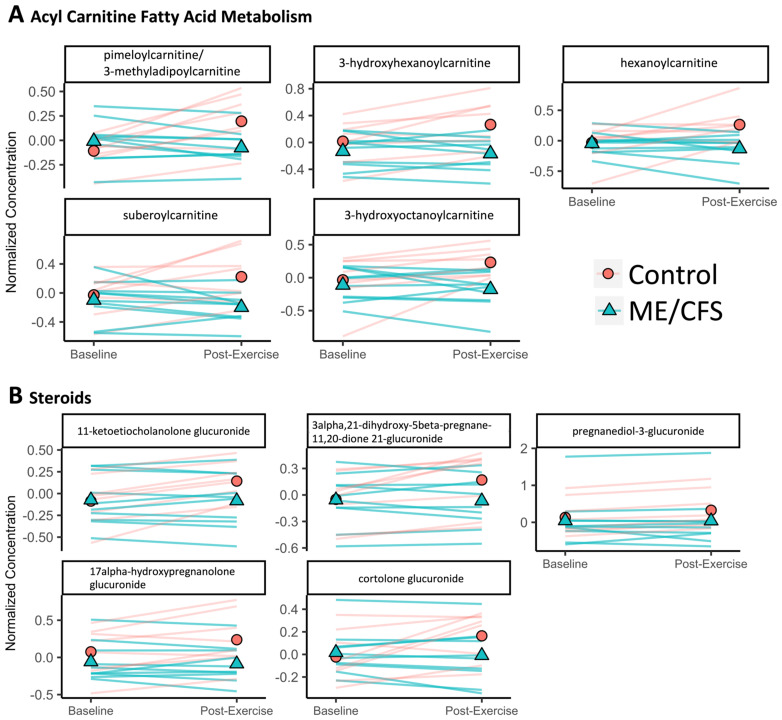
**Select lipid compounds changing differently during exercise recovery in ME/CFS patients vs. controls.** (**A**) Acyl carnitines. (**B**) Steroids. (**A**,**B**) Shown are all of the data points for ME/CFS and healthy controls for both time points, baseline and post-exercise. The red circle and the blue triangle represent the mean at each time point of the healthy controls and the ME/CFS patients, respectively. Each line is one subject. All compounds shown have *q* < 0.1 in the interaction term of the LMM, and are also significantly increasing in the controls from baseline to post-exercise (*q* < 0.1). The compounds within each section are in order of lowest to highest *q*-value.

The superpathways with the most altered compounds are amino acid and lipid, when considering the 110 compounds that have a significant interaction (*q* < 0.1) between disease status (ME/CFS vs. control) and time (baseline vs. post-exercise) (Supplementary Figure S2). Figure 6 shows the data for every subject in both groups at both baseline and post-exercise for the significantly altered compounds in several amino acid subpathways (Figure 6A–D). Figure 7 shows the data for all subjects at both baseline and post-exercise for the significantly altered compounds in the selected lipid subpathways (Figure 7A,B). For this figure, we combined the three subpathways involved in acyl carnitine fatty acid metabolism (dicarboxylate, hydroxy, and medium chain) and the four steroid subpathways (androgenic, cortico-, pregnenolone, and progestin). Every compound shown in Figure 6 and Figure 7 is also significantly increasing in urine post-exercise vs. baseline in the sedentary controls.

Four compounds in the urea cycle; arginine and proline metabolism subpathway are changing differently after exercise in the ME/CFS patients and controls: carboxy-methyl-arginine, proline, symmetric dimethylarginine (SDMA), and dimethylarginine (ADMA) (Figure 6A). Proline is a building block of collagen and is therefore a key component of connective tissues. SDMA and ADMA are both regulators and competitive inhibitors of nitric oxide (NO) production. NO aids in vascular maintenance in healthy individuals [33], and decreased NO production is associated with endothelial dysfunction and cardiovascular disease [34]. ADMA can be removed through urinary excretion or it can be degraded in the liver [35]. The increased excretion of SDMA and ADMA in controls but not in patients after exercise implies that controls may be removing excess NO synthase inhibitors in order to maintain vascular homeostasis and that this beneficial adaptation to exertion may not be occurring in patients. The relationship of NO and ME/CFS is unclear; plasma from ME/CFS subjects at baseline was found to induce less NO production by endothelial cells in vitro [36], but it is unknown whether or not that was due to higher levels of ADMA or SDMA in ME/CFS plasma, as they were not measured in that study and NO regulation is complex.

Three compounds in the methionine, cysteine, S-adenosylmethionine (SAM), and taurine subpathway are significantly altered: methionine sulfone, cysteine, and s-methylcysteine sulfoxide (Figure 6B). Cysteine is a unique amino acid in that it contains a thiol group and can participate in redox reactions [37]. Cysteine can also be converted into pyruvate, the starting point for the TCA cycle.

The polyamine metabolism subpathway has five significantly different metabolites: asicoga, (N(1)+N(8))-acetylspermidine, diacetylspermidine, N1,N12-diacetylspermine, and N-acetyl-isoputreanine (Figure 6C). Polyamines have a wide variety of biological functions and are involved in cellular proliferation, differentiation, and apoptosis [38]. An increase in polyamines is part of the normal response to stressors, including exercise [39].

The leucine, isoleucine, and valine metabolism subpathway also has five significantly altered metabolites: 3-methylglutarylcarnitine, tiglyl carnitine, valine, beta-hydroxyisovalerate, and beta-hydroxyisovaleroylcarnitine (Figure 6D). Leucine, isoleucine, and valine are the branch chain amino acids (BCAAs). These essential amino acids promote protein anabolism in human muscle which helps build muscle following exercise [40]. The catabolism of the three BCAAs leads to energy metabolism pathways and valine is glucogenic, meaning it is converted into glucose precursors which can enter the TCA cycle. While most amino acids are catabolized in the liver, BCAAs are mostly catabolized in other tissues including skeletal muscle, brain, kidney, and adipose tissue [41]. Isoleucine is both glucogenic and ketogenic, and leucine is ketogenic. These significantly different metabolites are produced during the catabolism of all three BCAAs. 3-methylglutarylcarnitine as well as 3-hydroxyhexanoylcarnitine (which is categorized as an acyl carnitine by Metabolon^®^, see Figure 7A) are produced as leucine is converted to acetyl-CoA and acetoacetate. During isoleucine degradation into acetyl-CoA or propionoyl-CoA, tiglylcarnitine is produced at two different steps. Beta-hydroxyisovalerate is produced at three different steps of the valine degradation pathway.

Five of the significantly altered compounds between ME/CFS patients and controls are involved in acyl carnitine fatty acid metabolism: pimeloylcarnitine/3-methyladipoylcarnitine, 3-hydroxyhexanoylcarnitine, hexanoylcarnitine, suberoylcarnitine, and 3-hydroxyoctanoylcarnitine (Figure 7A). Acyl carnitines play a key role in long-chain fatty acid β-oxidation, which is the primary mode of energy metabolism during aerobic exercise [42].

Five of the significantly altered compounds are classified as steroids (Figure 7B). 11-ketoetiocholanolone glucuronide is an androgenic steroid. 3alpha,21-dihydroxy-5beta-pregnane-11,20-dione 21-glucuronide and cortolone glucuronide are corticosteroids. 17alpha-hydroxypregnanolone glucuronide is a pregnenolone steroid. pregnanediol-3-glucuronide is a progestin steroid. Glucuronides are produced in the liver to aid in excretion of substances by making them more water soluble. Corticosteroids function as signaling molecules in a variety of processes, including promoting protein catabolism during exercise or other stressors [43], in mediating responses to inflammation [43], and in maintaining healthy salt and fluid levels [44]. Altered corticosteroid metabolism could be contributing to orthostatic intolerance, another ME/CFS symptom. Progestin steroids, androgenic steroids, and corticosteroids were also found at lower concentrations in female ME/CFS patient plasma compared to controls in another study, although that study investigated baseline levels only [11].

### 2.7. The Same Metabolites in Urine and Plasma Are Highly Correlated

**Figure 8:**
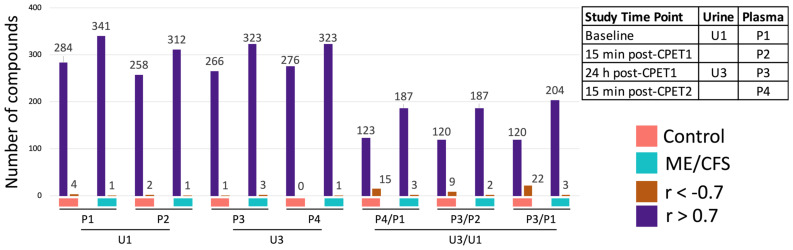
**Number of compounds with strong correlations between urine and plasma for the same metabolite.** Shown are the number of compounds with correlation coefficients (Pearson’s R) above 0.7 or below −0.7 out of 727 metabolites for which correlations were analyzed for each time point comparison. The key in the top right shows definitions for each time point abbreviation. U1 and U3 were correlated with plasma samples obtained the same day (U1 with P1 and P2; U3 with P3 and P4). The U3/U1 ratio was correlated with three plasma ratios: P4/P1, P3/P2, and P3/P1.

Our group previously published plasma metabolomics data from these same subjects [25]. These subjects underwent the complete two-day CPET protocol and along with urine collection, blood was drawn from each subject at four time points: baseline (P1), 15–30 min after the CPET (P2), 24 h after the CPET (P3), and 15–30 min after the second CPET (P4) which was performed 24 h after the first CPET (Figure 1A in [25]). Out of the 1403 urine metabolites and 1157 plasma metabolites detected by Metabolon^®^’s platforms, 727 compounds were measured in both urine and plasma. The relationship between the urine and plasma metabolomes as well as the influence of exercise on this relationship was evaluated by calculating the Pearson correlation coefficients^®^) between the urine and plasma datasets for each metabolite at all possible time point and time point ratio combinations (Supplementary File S3). For the time points, we chose to focus on the combinations of urine and plasma from the same day (baseline urine (U1) with P1 and P2, and post-exercise urine (U3) with P3 and P4). We also examined correlations between the post-exercise/baseline ratio in urine (U3/U1) and three different post-exercise ratios in plasma (P4/P1, P3/P2, P3/P1) to explore how the metabolite levels are changing during the 24 h recovery period in urine vs. plasma. The *p*-values were calculated for each correlation using a *t*-test with the null hypothesis of R = 0 (BH FDR correction, *q* < 0.15). The number of strong correlations, which we defined as R > 0.7 or R < −0.7, amounts to approximately 40% of the 727 metabolites when looking at time point correlations (Figure 8). Notably, for all time point pairs, there are very few strong negative correlations between urine and plasma. For the ratio correlations, the number of strong negative correlations is increased in the healthy controls. For all correlations, the number of strong positive correlations is higher in the ME/CFS patients than the controls.

### 2.8. Probing Compounds with Correlations between Urine and Plasma That Are Different in ME/CFS Patients and Controls

**Figure 9:**
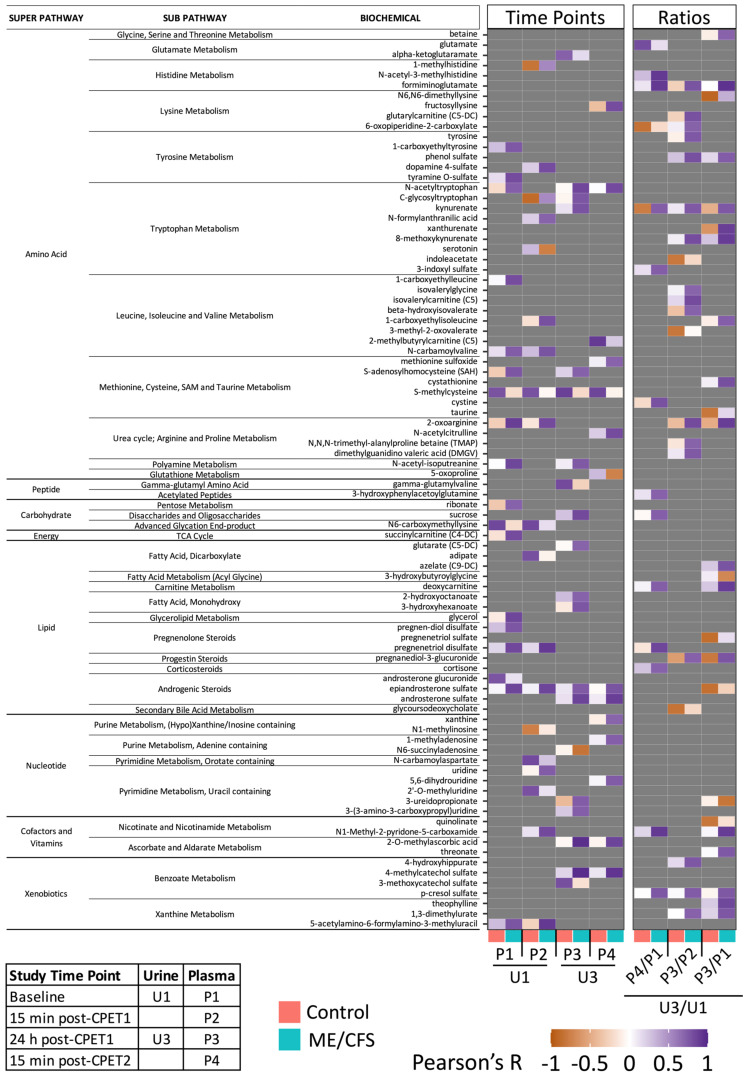
**Compounds with correlations between urine and plasma that are different in ME/CFS patients and controls.** The heatmap shows the correlation coefficient (Pearson’s R) in both ME/CFS and control groups for compounds and comparisons which meet the following criteria: (1) |R| > 0.7, *p* < 0.05, and *q* < 0.15 in either ME/CFS patients or controls; (2) R < 0.3 with the same sign or an R value with an opposite sign (i.e., negative if the significant correlation was positive) in the other cohort (controls or patients); (3) compounds that had extreme outliers were removed (modified z-score method of outlier detection, z > 6). Within each compound, the R values for the comparisons that did not meet the criteria are not shown (gray boxes). The left panel of the heatmap shows the time point comparisons and the right panel shows the ratio comparisons. The table shows the biochemical name and to which Metabolon^®^ subpathway and superpathway it belongs.

We proceeded to screen for compounds with the most significant differences between controls and patients, using the following stringent criteria: (1) |R| > 0.7, *p* < 0.05, and *q* < 0.15 in either ME/CFS patients or controls; (2) R < 0.3 with the same sign or an R value with an opposite sign (i.e., negative if the significant correlation was positive) in the other cohort (controls or patients); (3) compounds that had extreme outliers usually affecting the linear relationship were removed (modified z-score method of outlier detection, with a threshold of z > 6). When the outlier was only found in the time point data, that compound was removed for all but only the time point comparisons. When the outlier was found in the ratio data, that compound was removed for all but only the ratio comparisons. The summary of the compounds that met the above criteria is displayed as a heatmap of R values in Figure 9.

The heatmap contains metabolites spanning 35 subpathways with an overrepresentation of subpathways in the amino acid superpathway, 11 out of 15. Within the amino acid subpathways, tryptophan metabolism as well as leucine, isoleucine, and valine metabolism, had the most affected compounds, with 9/21 (43%) and 8/27 (30%), respectively.

**Figure 10:**
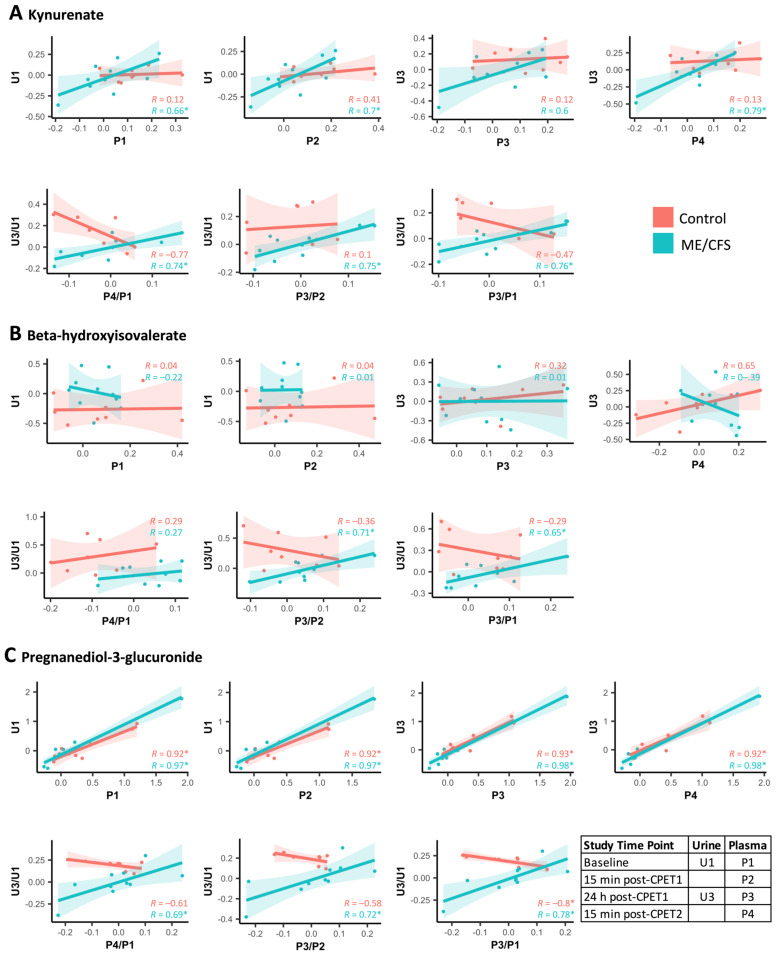
**Correlations between urine and plasma for all comparisons for select compounds from Figure 9.** Each dot is one subject. The lines are linear regression lines for each group, ME/CFS or control, and the shaded regions show the 95% confidence intervals. Pearson’s R is shown on each plot for controls (red) and ME/CFS patients (blue). * indicates correlations that are significant (*p* < 0.05 and *q* < 0.15). The time points are defined in the key in the bottom left.

Kynurenate is part of the tryptophan pathway and is one the metabolites with the most drastic difference in correlation coefficients between the ME/CFS and the control cohorts (Figure 9, P4/P1 with U3/U1). The kynurenate correlation graphs for all comparisons from the heatmap of Figure 9 are shown in Figure 10A. We can clearly see the inverted correlations in the “P4/P1 with U3/U1” graph where R = 0.74 (ME/CFS) and R = −0.77 (controls). The strong positive correlation in the ME/CFS patients is consistent throughout the ratio correlations, whereas the negative correlation in the controls is not as consistent. The time point correlations show a similar pattern, with a strong positive correlation for the ME/CFS group. While kynurenate is the only tryptophan compound with correlations in both the time point and the ratio sides of the heatmap, the differences seen in plasma and urine correlations in the other eight compounds, which appear at various locations in the tryptophan pathway, attest to a profound dysregulation of this pathway in the ME/CFS patients compared to the controls. Additionally, another compound on the heatmap, quinolinate, is the metabolite that links tryptophan metabolism to nicotinate and nicotinamide metabolism, which is a crucial pathway for the formation of NAD+ and NADP+. A dysregulation in the kynurenate pathway has been hypothesized to be the underlying cause of ME/CFS pathophysiology due to its central role in cellular energy production and involvement in mediating the immune response as reviewed by Kavyani et al. [45].

Eight compounds in the leucine, isoleucine, and valine subpathway had differences in the correlations between ME/CFS patients and controls for the selected time point and ratio comparisons (Figure 9). For five of these compounds, the differences were in the post-exercise ratios, with four out of the five compounds having differences when correlating the U3/U1 (24 h post-exercise/baseline urine) with P3/P2 (the 24 h post-exercise/15 min post-exercise plasma). Beta-hydroxyisovalerate is one such compound, with a strong and significant positive correlation in the ME/CFS patients between U3/U1 and P3/P2 and a weak, non-significant negative correlation in the controls (Figure 10B). Beta-hydroxyisovalerate is also changing significantly differently over time in the urine in the ME/CFS patients compared to the controls in the LMM (Figure 6D). These eight compounds span all three branches of the BCAA catabolism pathway. Isovalerylglycine and isovalerylcarntine are produced during leucine catabolism (ketogenic). 2-methylbutrylcarnitine and 3-methyl-2-oxovalerate are produced during isoleucine catabolism (ketogenic and glucogenic). Beta-hydroxyisovalerate, as mentioned above, is downstream of valine catabolism (glucogenic). This is further evidence that there is dysfunctional metabolic recovery from exercise in the ME/CFS patients related to BCAA catabolism, which is affecting all three BCAAs. The three BCAAs have a common enzyme involved in the first step of the pathway, BCAA aminotransferase. However, considering that there is dysregulation in so many amino acid subpathways, it is likely that this is evidence of a more global metabolic problem.

Within the lipid superpathway, four subpathways pertaining to steroids also caught our attention. Indeed, eight steroid compounds from four subpathways had different correlations between plasma and urine in the ME/CFS patients and controls, including androgenic steroids, corticosteroids, pregnenolone steroids, and progestin steroids (Figure 9). Pregnanediol-3-glucuronide, which is a progestin steroid and a product of progesterone catabolism, has a strong and significant positive correlation (R = 0.78) between U3/U1 and P3/P1 in the ME/CFS patients and a strong and significant negative correlation in the healthy controls (R = −0.8) (Figure 10C). In the ME/CFS patients, when pregnanediol-3-glucuronide increases in plasma 24 h post-exercise, it also increases in the urine and vice versa. Whereas in the healthy controls, the subjects with the largest increases in urine concentration of pregnanediol-3-glucuronide 24 h post-exercise have a decrease in plasma levels. This same trend is seen in the urine and plasma correlation for the other ratio comparisons. This compound is also changing significantly differently in the LMM between ME/CFS patients and controls, where the controls have a consistent post-exercise increase in urine concentration that is not seen in the ME/CFS patients (Figure 7B). At all four time point comparisons, the plasma and urine levels of pregnanediol-3-glucuronide are highly correlated in both groups of subjects, which has been shown before [46]. It is only when examining the change over time after exercise that the differences between the ME/CFS patients and healthy controls emerge. Although pregnanediol-3-glucuronide levels are not reported in acute exercise studies, it has been measured over the course of menstrual cycles in exercising vs. sedentary females, and the exercising females typically have lower urinary levels overall compared to sedentary females [46,47]. Given our results, it is possible that acute exercise initially leads to an increase in urinary pregnanediol-3-glucuronide levels in healthy sedentary females as they are excreting it and not replacing it. This healthy response to exercise is not occurring in the ME/CFS patients, which is yet further evidence for their overall altered metabolic response to exercise.

We generated another heatmap that contains unknowns, partially characterized molecules and food components meeting the same criteria used to generate Figure 9 (Supplementary Figure S5). This is provided as additional information to illustrate the potential of some yet to be identified metabolites. As an example, X–25524 consistently shows strong positive correlation for the ME/CFS group but no correlation for the control group regardless of exercise. Identifying such a compound could potentially help develop a diagnostic marker for ME/CFS by measuring blood and urine concentrations.

## 3. Discussion

This is the first time that the urine metabolome of ME/CFS patients has been characterized before and after an exercise challenge, when ME/CFS patients are experiencing PEM. Many of these metabolites have never before been measured in ME/CFS patients, since previous urine metabolomics studies in ME/CFS have been limited to less than 50 metabolites and the current study measured 1403. Moreover, the use of sedentary healthy controls to account for physical activity level, which can affect baseline and post-exercise metabolite levels, is a key advantage of the current study design that has not been utilized in previous studies. Our results showed widespread increases in the levels of metabolites in the urine of the controls 24 h post-exercise that were not seen in the ME/CFS patients, with 110 of these compounds having a significant interaction between disease status (ME/CFS or control) and time (baseline vs. post-exercise) (Supplementary Figure S2). In addition to numerous analyses of urinary metabolite levels, correlating metabolite levels in urine and plasma yielded additional evidence of metabolic dysregulation in the ME/CFS patients post-exercise. This analysis provided further evidence of pathophysiological changes in multiple subpathways as well as evidence of differences in additional subpathways that did not have many significant differences between the ME/CFS patients and controls when looking at urine metabolite levels in isolation.

### 3.1. Comparison to Previous Urine Metabolomics Studies in ME/CFS Patients

Overall, our results are not consistent with the few previous studies measuring urine metabolites in ME/CFS patients compared to control non-ME/CFS subjects. To better compare our results with previous studies, which measured fewer metabolites, we compared the results at baseline for *p* < 0.05 in the LMM to the previous studies. The only compound that was found to be significant in another study and ours was alanine, although the previous study found alanine to be lower in female patients than in controls (BH-adjust *p*-value < 0.05) and in our study the mean normalized concentration was higher in the ME/CFS patients than in controls [20]. However, several of the studies found differences at baseline in compounds that we found were changing differently in the ME/CFS patients and controls during exercise recovery, including phenylalanine (lower in ME/CFS patients [23,24]) and valine (lower in ME/CFS patients [20]). Both phenylalanine and valine were also significantly increased in the sedentary controls following exercise in the current study, so it is possible that the controls in other studies were more active and already had higher levels of urinary phenylalanine. No other studies specifically recruited sedentary non-ME/CFS subjects, although one study did seek to match “general lifestyle” [23]. Armstrong et al. looked at Pearson’s correlations between urine and plasma metabolites in ME/CFS patients and controls at baseline, and found differences in acetate, lactate, and phenylalanine with a threshold of |R| > 0.4 in either group [20]. Acetate is too small to be detected in our assay and we did not detect differences in plasma and urine correlations in lactate nor phenylalanine.

McGregor and colleagues also investigated changes in the urine and plasma metabolomes in ME/CFS patients experiencing PEM [19]. They used a survey to separate ME/CFS patients currently experiencing PEM in the last seven days and discovered that eight out of thirty urine metabolites measured had significantly lower concentrations in the ME/CFS group compared to the controls. Of these, only serine had significant differences in any of our analyses; it increased after exercise in the control group (Supplementary Data File S2—LMM Results). Levels of two urine metabolites, acetate and methylhistidine, were also significantly different in the PEM vs. the no PEM group [19]. Levels of the methylhistidines assayed in this study were not significantly different in the LMM analysis, but we did find differences in the plasma and urine correlations of 1-methylhistidine and N-acetyl-3-methylhistidine (Figure 9). McGregor et al. also found associations of seven-day PEM scores with several metabolites in plasma and urine [19].

### 3.2. The Post-Exercise Increase in Urinary Metabolite Levels in Sedentary Controls Is Consistent with Previous Studies

The urine metabolome in females 24 h post-exercise has not been well characterized. To the best our knowledge, no studies have measured the urine metabolome at baseline compared to 24 h post-exercise in females. One study measured 32 metabolites in urine before exercise and 24 h post-exercise in men, comparing nine competitive cyclists to eight healthy but untrained men of the same age (50–60 years old) [48]. While their study focused on comparing the athletes to the untrained subjects, they did see high fold change increases post-exercise (greater than two-fold) in the control subjects in lactate, acetate, and hypoxanthine levels. Acetate was not measured in our study, and neither lactate nor hypoxanthine were different from baseline to post-exercise in our female control group. Mukherjee et al. did find significant differences between the athlete and control groups in eight of the measured metabolites linked to a variety of biochemical pathways [48]. Therefore, a strength of the current study is the selection of sedentary healthy controls as opposed to more active individuals, who may have an altered urine metabolome due to regular exercise.

While there is a dearth of published literature on the urine metabolome 24 h post-exercise, there are several studies measuring urine metabolites in both males and females at earlier post-exercise time points (reviewed in [49]). One of the findings which was consistent between studies is that the concentration of most lipids increases in biofluids post-exercise, including in urine. In particular, acyl carnitine concentrations have been shown to increase in blood and urine in response to exercise. This is consistent with the results of our study in which several acyl carnitine compounds were significantly increased post-exercise in the urine of the controls (Figure 7A).

The largest study which included females (255 total subjects, 107 female) also found extensive metabolic changes in urine post-exercise, with 37 out of 47 measured metabolites significantly altered after FDR correction, and 33 of those were increased post-exercise [50]. This is consistent with our finding of large-scale metabolic change post-exercise in the urine of control subjects, with the majority of the compounds that were altered found to have increased concentrations. This study also completed a sex-stratified comparative analysis but only found two metabolites with significantly different post-exercise/baseline ratios in females and males.

In the Schranner et al. review, the findings for amino acids are not as consistent as those for lipids, which generally increase post-exercise [49]. However, there were some findings in urine that were consistent across at least two studies (although all post-exercise time points are combined), including that the following compounds increased in urine post-exercise: alanine, O-acetyl-homoserine, 5-hydroxyindolepyruvate, xanthurenate, L-metanephrine, N-acetylvanilalanine, and N-(carboxyethyl) arginine. The following compounds were found to be decreased in urine post-exercise in at least two studies: glycine, histidine, trimethylamine n–oxide. Comparing these results to our study, most of the metabolites were either not significantly different pre- and post-exercise, or were not measured in our study. However, we also found a significant increase in alanine levels in the controls, which is consistent with the studies reviewed. In our study, glycine levels were also increased post-exercise in controls as opposed to decreased. However, the Kistner et al. study, which included a large number of females, also found glycine levels to be significantly increased post-exercise [50].

### 3.3. Differences between Sedentary Controls and ME/CFS Patients in the Lipid Superpathway

Many lipid subpathways were significantly different in the urine of the patients and controls in this study, including acyl carnitine fatty acid metabolism. Acyl carnitine metabolites were increased post-exercise in the urine in healthy controls and the changes induced by exercise were significantly different between the controls and ME/CFS patients (Figure 3, Figure 7 and Figure S4). Additionally, although not an acyl carnitine, deoxycarnitine in the carnitine metabolism lipid subpathway correlated differently between plasma and urine in the ME/CFS patients compared to controls (Figure 9). Acyl carnitines are very important in energy metabolism, as they are required to transport fatty acids into the mitochondria for β-oxidation. Long-chain fatty acid β-oxidation is the primary mode of energy metabolism during aerobic exercise. Disrupted acyl carnitine metabolism during exercise could be contributing to exercise intolerance and PEM in ME/CFS patients. In another study looking only at subjects at baseline and that did not specifically recruit sedentary controls, the acyl carnitine subpathway was found to be significantly different in ME/CFS patients vs. controls, with five of eight compounds found to have a lower concentration in the patients [11]. When only baseline subjects were analyzed, specific measurements of acyl carnitine in serum indicated that the compound was lower in ME/CFS patients than the controls in one report [51] but no differences in urine or plasma levels were seen in another study [52]. In the plasma of the larger cohort of which the subjects of the current study are a subset, the carnitine chemical cluster was also significantly altered in female sedentary controls during recovery (defined as the difference between 24 h post-exercise and 15 min post-exercise) with the majority of the compounds increasing post-exercise [25]. The carnitine chemical cluster was not found to be significantly altered during exercise recovery in ME/CFS patients. While this cluster does include more than just acyl carnitines, acyl carnitines are members and are contributing to its significance in chemical similarity enrichment analysis in the current study as well (Supplementary Figure S4). It has also been shown ex vivo that palmitoylcarnitine, which is increased in muscle transiently post-exercise, may act as an exertion signal from muscle to a subset of neurons [53].

Acyl glycine fatty acid metabolites are the only compounds that were found in urine at significantly different concentrations in ME/CFS vs. controls at a single time point (24 h post-exercise) and a different acyl glycine compound, 3-hydroxybutyroylglycine, had a significant negative correlation in the ME/CFS patients when correlating U3/U1 with P3/P1 (Figure 5 and Figure 9). Additionally, cis-3,4-methyleneheptanoylglycine was changing differently during exercise recovery in the ME/CFS patients vs. controls (LMM, Supplementary Figure S2). While acyl glycine metabolism is not one of the subpathways that was significantly increased post-exercise in the controls alone, it was significantly different in the ME/CFS patients vs. controls both at the 24 h post-exercise time point and when analyzing the difference in the post-exercise/baseline ratios (Figure 3). Urinary excretion of particular acyl glycines is also altered by disorders linked to fatty acid β-oxidation in the mitochondria, including medium-chain acyl-coenzyme A (CoA) dehydrogenase (MCAD) deficiency [54]. Our group has observed that fatty acid oxidation differs in immune cells from ME/CFS patients vs. controls [55].

### 3.4. Differences between Sedentary Controls and ME/CFS Patients in the Amino Acid Superpathway

We also found many differences in urine in amino acids in the ME/CFS patients and controls post-exercise. Two of those pathways stood out because they had significant alterations in the ME/CFS patients vs. controls in all of our analyses, including the KEGG pathway analysis, and are discussed further below.

The urea cycle in the liver is an important part of exercise metabolism because it is needed to remove high levels of ammonia that are produced during exercise [56,57]. Germain and colleagues also found that the urea cycle and the ammonia recycling SMPDB pathways were significantly altered in the plasma between ME/CFS female patients and controls in a pathway analysis when comparing the difference between metabolite levels at 24 h post-CPET (P3) and 15 min post-CPET (P2) [25]. Ammonia buildup has been previously linked to neurotoxicity and exercise-induced fatigue [56,57]. It is possible that the dysregulation of the urea cycle in the urine and plasma metabolomes after exercise in ME/CFS patients is causing ammonia buildup, but the 1403 compounds measured by Metabolon^®^ in the urine did not include ammonia because it is a volatile compound and also smaller than the detection limit of Metabolon^®^’s platform.

Cysteine, methionine, SAM, and taurine are important amino acids as they are the only ones that contain sulfur, and cysteine is unique in its ability to form disulfide bonds. Cysteine may also be converted into glutathione and taurine. Cysteine and methionine play numerous roles in cellular metabolism but they are also key building blocks of proteins [37]. Because of its thiol group, cysteine is involved in catalyzing many enzymatic reactions and maintaining redox homeostasis. Changes in cysteine metabolism occur in many neurodegenerative disorders, including Alzheimer’s disease, Huntington’s disease, and Parkinson’s disease [58]. While cysteine, methionine, SAM, and taurine metabolism showed many differences between the patients and controls in our urine metabolome analyses, the urine and plasma correlations revealed additional compounds with significant differences between the ME/CFS patients and controls, including in cystine, which is produced when two cysteines are oxidized to form a disulfide bond, and cystathione which is an intermediate in cysteine production in the methionine cycle [37].

### 3.5. Limitations

Our study has several important limitations. First, the diet of the subjects was not controlled, and dietary intake of metabolites can affect their excretion in urine. Second, we acknowledge that the lack of BMI matching is not ideal and is a limitation of this study. Our larger cohort of ME/CFS patients and healthy sedentary controls is BMI-matched, and therefore if this pilot study is expanded, this will not be an issue in the future. Third, our results are limited to female ME/CFS patients. While it is very important to study both sexes in ME/CFS and an increasing number of sex differences in pathophysiology are being discovered [25,59,60], we chose to focus our pilot study on females because of the higher disease burden of ME/CFS in females (60–65% female) [2]. Additionally, because we captured the urine metabolome only at two time points, baseline and 24 h post-exercise, we cannot say whether or not the ME/CFS patients have altered excretion levels of some of these metabolites at either an earlier or later time point than the controls. It is possible that these increases in excretion products are happening in patients but with a larger delay, similar to how the ME/CFS patients show a delayed overall recovery to exercise. However, it is also possible that this lack of altered metabolic excretion is part of an overall lack of a healthy metabolic response to exercise.

## 4. Materials and Methods

### 4.1. Study Subjects

Eight healthy sedentary controls and ten ME/CFS patients were included in this study. ME/CFS patients were diagnosed with the Canadian Consensus Criteria [3]. The 18 subjects included in this study were part of a larger cohort of 173 participants total (ClinicalTrials.gov Identifier: NCT04026425) [61]. For this pilot study, all subjects included were female. Subjects were recruited with the following criteria. All participants must be between 18–70 years old. Subjects were excluded from either group if they were a smoker, pregnant or breastfeeding, were diabetic, consumed excessive amounts of alcohol, or had an orthopedic limitation preventing them from performing the CPET. Diagnoses of schizophrenia, major depressive disorder, bipolar disorder, or an anxiety disorder were also exclusion criteria in both groups. Additionally, healthy sedentary controls were excluded if they were diagnosed with any autoimmune disorders. Renal function was normal in all of the subjects for this study, as assessed by the following Quest Diagnostics standard laboratory blood tests: serum creatinine, blood urea nitrogen, and estimated glomerular filtration rate (eGFR).

Seventeen subjects performed the exercise testing at Ithaca College in Ithaca, New York and one subject performed the exercise testing at ID Med in Torrance, California. All participants were asked to stop nutritional supplements including probiotics for two weeks prior to exercise testing. Participants were asked to stop pain and stimulant medication for two days prior to the exercise testing. All patients provided written informed consent, and all protocols were approved by Ithaca College IRB #1017-12Dx2. All participants completed the Bell Disability Scale [26], Short Form-36 health survey [62], and custom questionnaires. The ME/CFS patients additionally completed the multidimensional fatigue inventory [63].

### 4.2. Cardiopulmonary Exercise Testing and Urine Sample Collection

The CPET was performed on a stationary cycle ergometer, with the following protocol: 3 min of rest followed by continuous cycling in which the incremental workload increases 15 watts per minute of exercise until volitional exhaustion (approx. 8–10 min). The respiratory exchange ratio (RER), which is the rate of carbon dioxide production divided by the rate of oxygen consumption, was measured to ensure that participants were performing the test with sufficient effort (RER > 1.1 indicates maximal effort).

All urine samples were collected in the morning: (1) 15–20 min prior to the CPET and (2) 24 h later. Urine was collected mid-stream in sterile urine collectors, aliquoted, centrifuged at 10,000× *g* for 10 min to remove cell debris, and stored at −80 °C. Urine samples underwent one freeze/thaw cycle for further aliquoting and the aliquots were shipped overnight to Metabolon^®^ on dry ice.

### 4.3. Metabolomics Assay

Metabolites were measured using the Precision Metabolomics™ liquid chromatography–tandem mass spectrometry (LC-MS/MS)global metabolomics platform at Metabolon^®^. Detailed methods have been described previously [64]. Briefly, samples were extracted in methanol (5:1 methanol:sample) and then evaporated. Metabolites were detected in each sample using four different LC-MS/MS platforms that were optimized for hydrophilic and hydrophobic compounds and using both positive and negative ionization. All chromatography utilized a Waters Acquity ultra-high performance (UP)LC and a 5 µL injection volume (with samples reconstituted in appropriate solvents for each platform). All mass spectrometry was performed with a ThermoScientific Q-Exactive high resolution/accurate mass spectrometers with heated electrospray ionization (HESI-II) sources and Orbitrap mass analyzers operated at 35,000 mass resolution with scan range 70–1000 *m*/*z*. Metabolon^®^ proprietary software was used to match experimental samples with a reference library of Tier 1 identification standards as defined by the Metabolomics Standards Initiative, and the area under the curve was used for peak quantification. Values are normalized in terms of raw area counts, and all samples were run in one batch so no batch correction was necessary. The unknown compounds do not have a standard, and partially characterized molecules are those that have not been officially confirmed based on a standard or for which a standard is not available, but Metabolon^®^ is reasonably confident in its identity.

### 4.4. Data Processing

Raw data were normalized by osmolality for each sample and the data for each metabolite were median-centered to 1 (raw data including osmolality are available in Supplementary File S1). Missing values were imputed with the minimum value, except for drugs which were imputed as 0. Data were log10 transformed with a variance stabilizing transformation (MetaboanalystR) [65,66]. A total of 1403 metabolites were originally measured. Metabolites were filtered according to the modified 80% rule: a compound was included if it was detected in at least 80% of the samples in either of the ME/CFS or control groups [27]. Overall, 1154 metabolites met the criteria and were included in subsequent analyses. The only analysis carried out without filtering was on the correlations with plasma metabolites. The post-exercise/baseline ratios for each metabolite were calculated in log base 10 as the post-exercise value minus the baseline value for each subject. For plotting on the volcano plot, the mean log10 fold changes (ME/CFS patients vs. controls) were converted to log base 2 using the change of base formula.

### 4.5. Data Analysis and Statistics

Univariate statistical analysis for each metabolite was performed using a linear mixed model with fixed effects of disease status, time point, age, and BMI and a random effect of subject (lmertest [67] and emmeans [68] R packages). The Benjamini–Hochberg (BH) method was used to correct for false discovery rate, with *q* < 0.1 used as the threshold for significance. The EnhancedVolcano R package was used for volcano plots [69].

ChemRICH in R was used to perform the non-overlapping pathway analysis with the Metabolon^®^-defined subpathways and the pathway order [29]. The ChemRICH webtool was used to perform the chemical similarity clustering analysis [30]. For that analysis, only compounds that had a known SMILES code were able to be included, for a total of 516 compounds. For both ChemRICH analyses, the enrichment statistics were performed using the Kolmogorov–Smirnov test, which does not use a *p*-value significance cutoff but rather compares the probability distribution with a null hypothesis probability distribution [70]. For the Metabolon^®^ subpathways, *q* < 0.05 was selected as the threshold for significance and *q* < 0.15 was selected for the chemical clusters (BH FDR correction). For both, all clusters below the *q* thresholds chosen also had *p* < 0.05.

Pathway enrichment and topology analysis was performed using the Metaboanalyst 5.0 web tool [65], for both the KEGG and SMPDB human reference metabolomes with the following parameters selected: global test for the statistics test and relative betweenness centrality as the node importance measure. Compounds were included in this analysis if the HMDB ID provided by Metabolon^®^ matched the HMDB ID in Metaboanalyst. For duplicate compounds for one HMDB ID, only the first one was included. This resulted in 453 included compounds.

The clustering of the subjects using the four compounds that were significantly different between the patients and controls post-exercise was performed using hierarchical clustering, with the Euclidean distance as the distance metric, and the method “Ward.D2” (pheatmap R package [71]).

Pearson correlations between urine and plasma for 727 metabolites measured in both biofluids were performed in R (hmisc package). *p*-values were calculated for each correlation using a *t*-test with the null hypothesis that the correlation coefficient equals 0, followed by BH FDR correction with *q* < 0.15 as the threshold for significance. For Figure 8, compounds were screened to remove those which had extreme outliers using the modified z-score method, which calculates a z score using the median and median absolute deviation (outliers R package, z threshold = 6).

Unless otherwise specified, all data visualizations were performed using the ggplot2 R package. BH FDR correction was chosen for all analyses instead of the more stringent Benjamini and Yekutieli FDR correction because an extremely small number of compounds were found to be colinear (0.75% of targets had an absolute value Pearson’s correlation coefficient > 0.7).

## 5. Conclusions

Overall, there were significant differences in the urine metabolome in the healthy sedentary controls and the ME/CFS patients in response to a CPET challenge in a large range of metabolic super and subpathways, spanning amino acids, lipids, carbohydrates, nucleotides, xenobiotics, and unknowns. These pathways are involved in a multitude of physiological functions including but not limited to energy metabolism. This indicates that ME/CFS patients have a general metabolic dysregulation that is part of their exercise intolerance and PEM in which altered metabolic excretion is a contributing factor. Our data suggest that the metabolisms of sedentary individuals who do not have ME/CFS undergo major changes that allow them to recover from exertion, while ME/CFS patients fail to make similar adaptive responses. Future work will include expanding this study to a much larger cohort that includes both sexes to validate these results, examine sex differences in the urine metabolome, and explore whether there are more subtle differences in urinary metabolites in ME/CFS patients at baseline that could potentially contribute to a diagnostic test for the disease in the future.

## Figures and Tables

**Table 1:** Study demographics.

	ME/CFS		Controls		
	Median	IQR	Median	IQR	*p*-Value
Age (years)	51.5	5.3	52.5	6.5	0.96
BMI	24	10.9	33.3	8.2	0.03 *
Bell disability scale	30	15	90	22.5	0.0004 *
SF-36 physical component summary (PCS)	25.6	7	54.5	7.1	0.00005 *
SF-36 mental component summary (MCS)	48	4.6	57	6.2	0.07
Multidimensional fatigue inventory (MFI)	83	10.3	NA	NA	NA
ME/CFS duration (years)	7.5	7.3	NA	NA	NA

Shown are the median and interquartile range (IQR) for demographic parameters in ME/CFS patients and control subjects. For the Bell disability scale and SF-36, a higher number corresponds to better health. For the MFI total score, a higher number corresponds to increased fatigue. The *p*-values are from a Wilcoxon rank sum test. * indicates a significant difference between the ME/CFS and the control groups (*p* < 0.05). NA: not applicable.

## Data Availability

All metabolite data for each subject are available in the provided Supplementary Data Files.

## Supplementary Materials

The following supporting information can be downloaded at: https://www.mdpi.com/article/10.3390/ijms24043685/s1.
