# Supplementary material for: Urine Metabolomics Exposes Anomalous Recovery after Maximal Exertion in Female ME/CFS Patients
Source: Int J Mol Sci. 2023 Feb 12;24(4):3685. doi: 10.3390/ijms24043685 (PMC9958671; doi:10.3390/ijms24043685)
Supplement: Supplementary file 1 [file ijms-24-03685-s001.zip › K.Glass Supplementary Figures_Final.pdf]

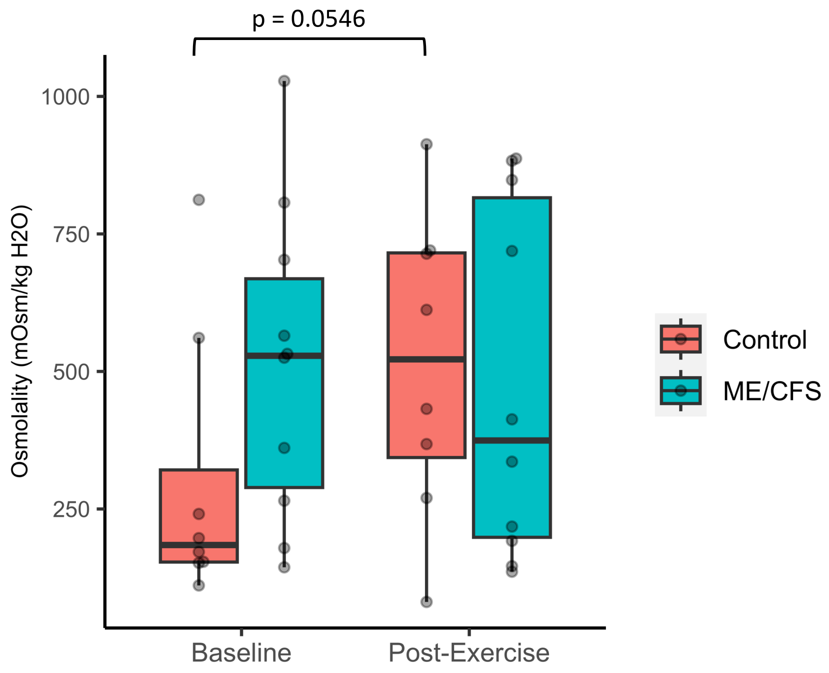

Supplementary Figure S1 – Urine osmolality (mOsm/kg H_2O) for controls and ME/CFS patients at baseline and post-exercise. A linear mixed effects model with the following formula was used to statistically compare the osmolality in the experimental groups: Osmolality ~ Disease status * Time Point + (1|Subject). Fixed effects were not significant but there was a significant interaction between disease status and time point ($p < 0.05$). Pairwise comparisons with Tukey's posthoc test were performed (*emmeans* package). No pairwise comparisons were significant but there was a trend towards increased osmolality post-exercise in the control group only ($p = 0.0546$).

Super Pathway	Subpathway	Biochemical
Amino Acid	Alanine and Aspartate Metabolism	hydroxyasparagine
	Glutathione Metabolism	2-hydroxybutyrate/2-hydroxyisobutyrate
	Glycine, Serine and Threonine Metabolism	2-methylserine
	Leucine, Isoleucine and Valine Metabolism	3-methylglutarylcarnitine (2)
		beta-hydroxyisovalerate
		beta-hydroxyisovaleroylcarnitine
		tiglyl carnitine (C5)
		valine
	Lysine Metabolism	saccharopine
	Methionine, Cysteine, SAM and Taurine Metabolism	cysteine
		methionine sulfone
		S-methylcysteine sulfoxide
	Phenylalanine Metabolism	phenylalanine
	Polyamine Metabolism	(N(1) + N(8))-acetylspermidine
		acisoga
		diacetylspermidine*
		N-acetyl-isoputreanine
		N1,N12-diacetylspermine
	Tryptophan Metabolism	C-glycosyltryptophan
	Urea cycle; Arginine and Proline Metabolism	dimethylarginine
		carboxy-methyl-arginine
		proline
		symmetric dimethylarginine (SDMA)
Carbohydrate	Aminosugar Metabolism	6-sialyl-N-acetyllactosamine
		N-acetylglucosamine/N-acetylgalactosamine
		N-acetylglucosaminylasparagine
	Pentose Metabolism	arabitol/xylitol
		arabonate/xylonate
		ribitol
Cofactors and Vitamins	Ascorbate and Aldarate Metabolism	gulonate*
Energy	TCA Cycle	homocitrate
Lipid	Androgenic Steroids	11-ketoetiocholanolone glucuronide
	Corticosteroids	3alpha,21-dihydroxy-5beta-pregnane-11,20-dione 21-glucuronide
		cortolone glucuronide (2)
	Fatty Acid Metabolism (Acyl Carnitine, Dicarboxylate)	pimeloylcarnitine/3-methyladipoylcarnitine (C7-DC)
		suberoylcarnitine (C8-DC)
	Fatty Acid Metabolism (Acyl Carnitine, Hydroxy)	3-hydroxyhexanoylcarnitine (1)
		3-hydroxyoctanoylcarnitine (1)
	Fatty Acid Metabolism (Acyl Carnitine, Medium Chain)	hexanoylcarnitine (C6)
	Fatty Acid Metabolism (Acyl Glycine)	cis-3,4-methyleneheptanoylglycine
	Fatty Acid, Dicarboxylate	3-methyladipate
		4-octenedioate
	Phospholipid Metabolism	glycerophosphoethanolamine
		glycerophosphorylcholine (GPC)
	Pregnenolone Steroids	17alpha-hydroxypregnanolone glucuronide
	Progestin Steroids	pregnanediol-3-glucuronide
	Secondary Bile Acid Metabolism	glycocholenate sulfate*
		glycoursodeoxycholic acid sulfate (1)
Nucleotide	Purine Metabolism, Adenine containing	adenosine 3',5'-cyclic monophosphate (cAMP)
		N6-succinyladenosine
	Pyrimidine Metabolism, Orotate containing	orotidine
	Pyrimidine Metabolism, Uracil containing	pseudouridine
Peptide	Dipeptide	phenylalanylhydroxyproline*
	Gamma-glutamyl Amino Acid	gamma-glutamylvaline
	Modified Peptides	N,N-dimethyl-pro-pro
Xenobiotics	Chemical	glycolate (hydroxyacetate)

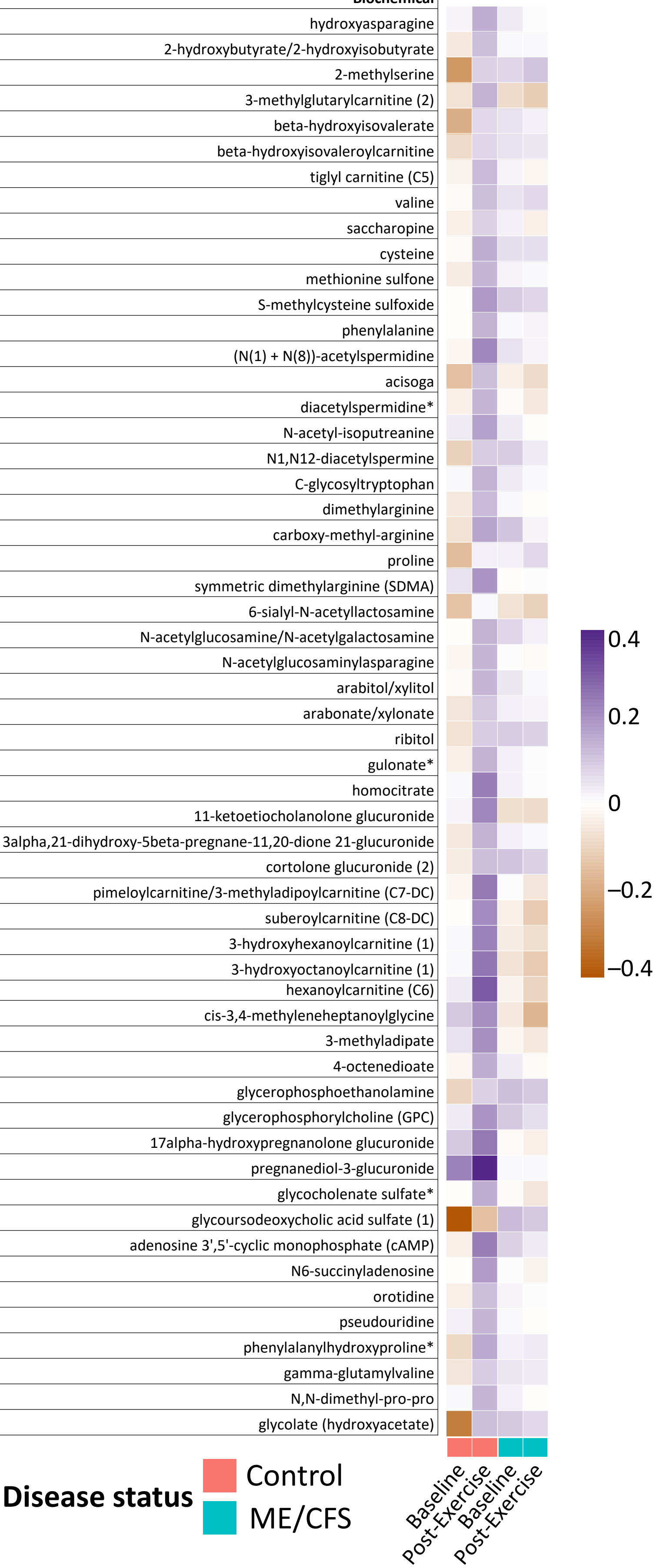

Supplementary Figure 2 – Compounds changing differently over time in ME/CFS patients and controls.

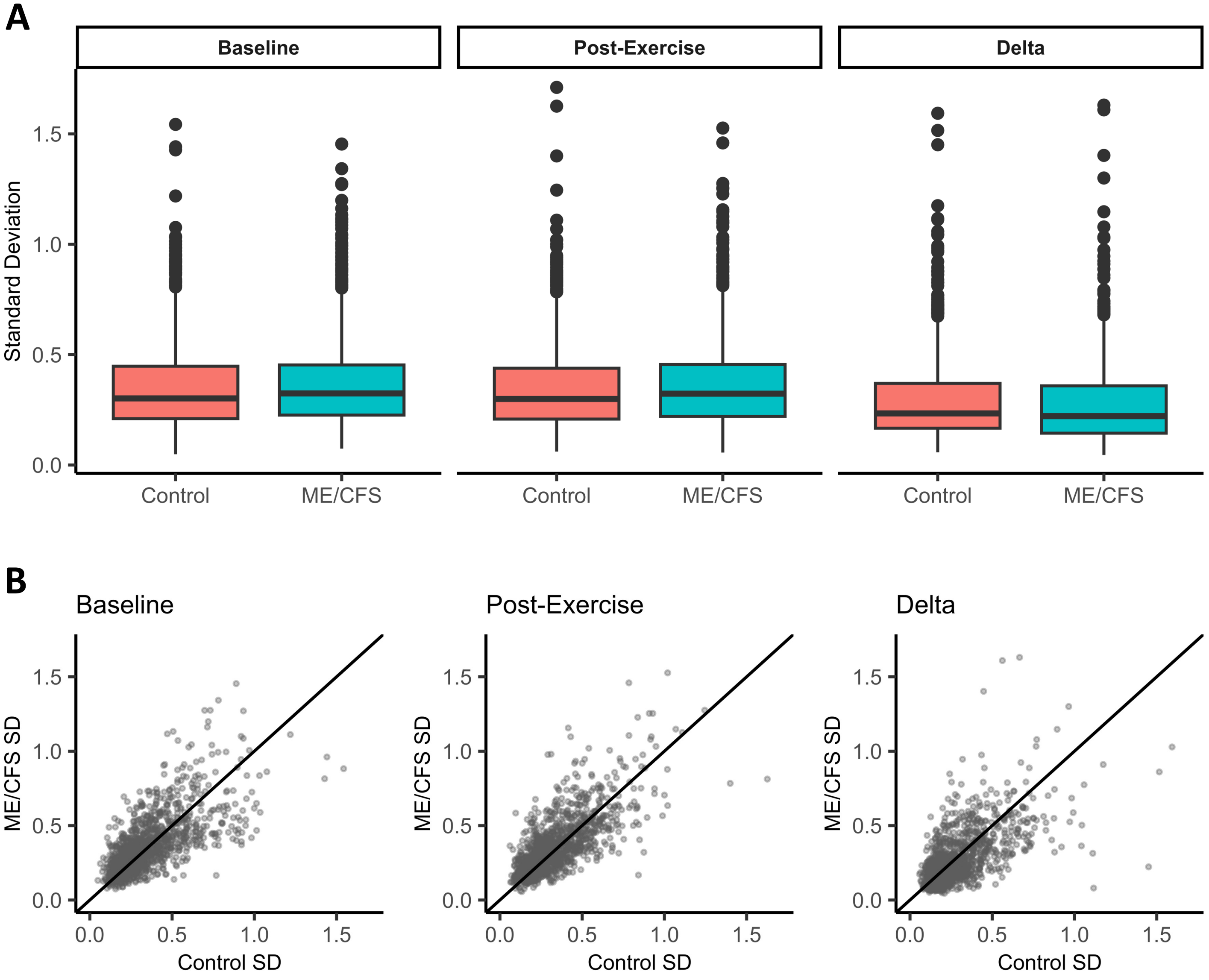

Supplementary Figure 3 – Standard Deviations for ME/CFS patients and controls. Shown are the standard deviations for the 1154 compounds analyzed, after missing values were imputed and log transformation. The "delta" dataset is the log2 fold change of post-exercise / baseline for each subject. **A.** Boxplots of all standard deviations (log10 scale). **B.** Scatterplots of the control SD vs the ME/CFS SD for each metabolite. Diagonal line at x = y.

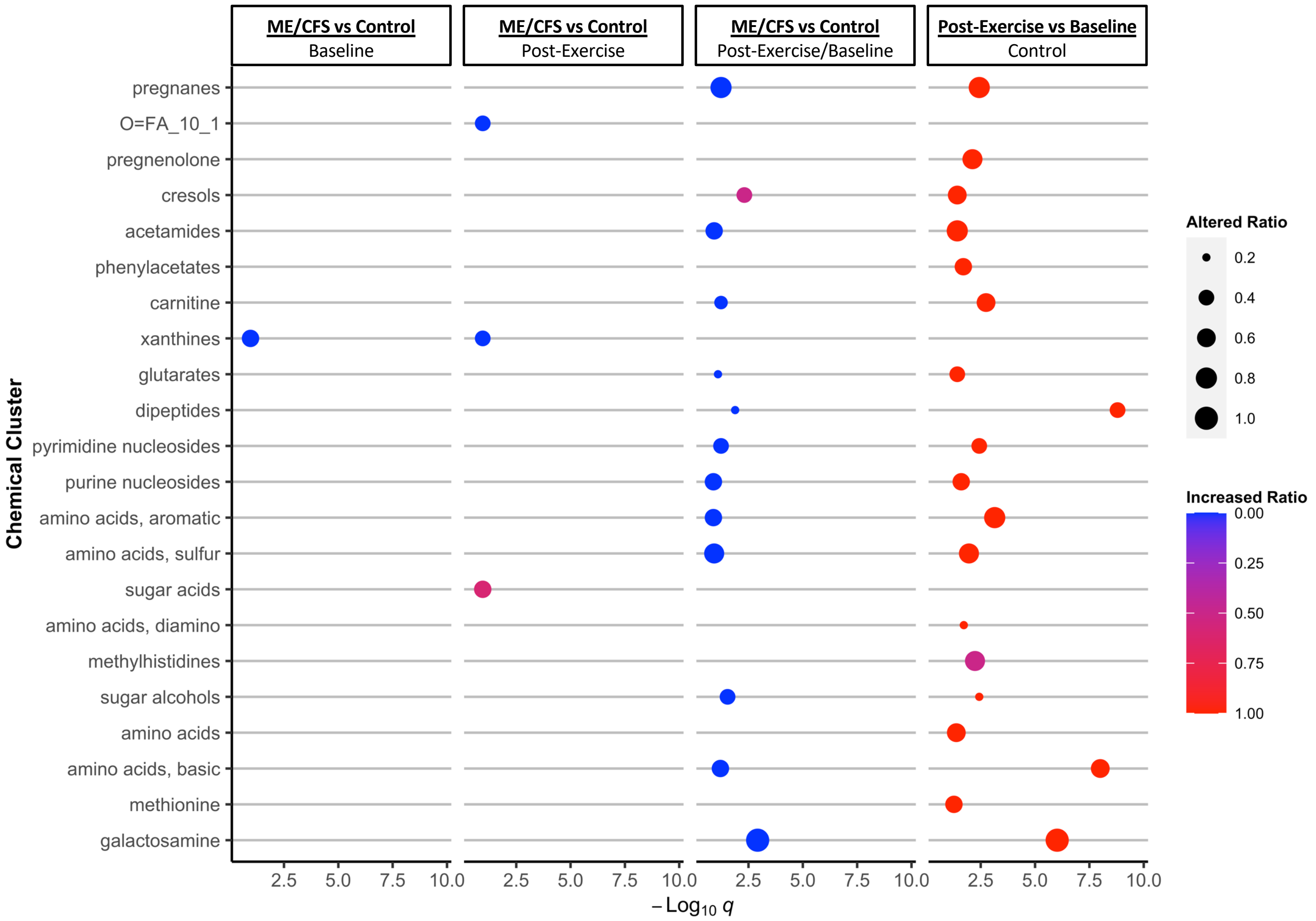

Supplementary Figure 4 – Chemical Similarity Enrichment Analysis. Shown are chemical clusters which were significantly altered for each comparison (Kolmogorov-Smirnov test, q < 0.15, BH FDR correction, and all clusters originally p < 0.025). The clusters are ordered by lipophilicity (non-polar at the top to polar on the bottom, median xlogP of all compounds in the cluster). The size of the bubbles shows the ratio of significantly altered metabolites to total metabolites in that cluster. The color gradient shows the increased ratio, where 0 indicates all of the altered metabolites were decreased in that comparison, and red indicates all altered compounds were increased. The post-exercise vs baseline comparison for the ME/CFS patients was also evaluated but no significantly altered chemical clusters were identified. 516 compounds assigned to 74 non-overlapping chemical clusters were included in this analysis. Analysis performed using https://chemrich.fiehnlab.ucdavis.edu.

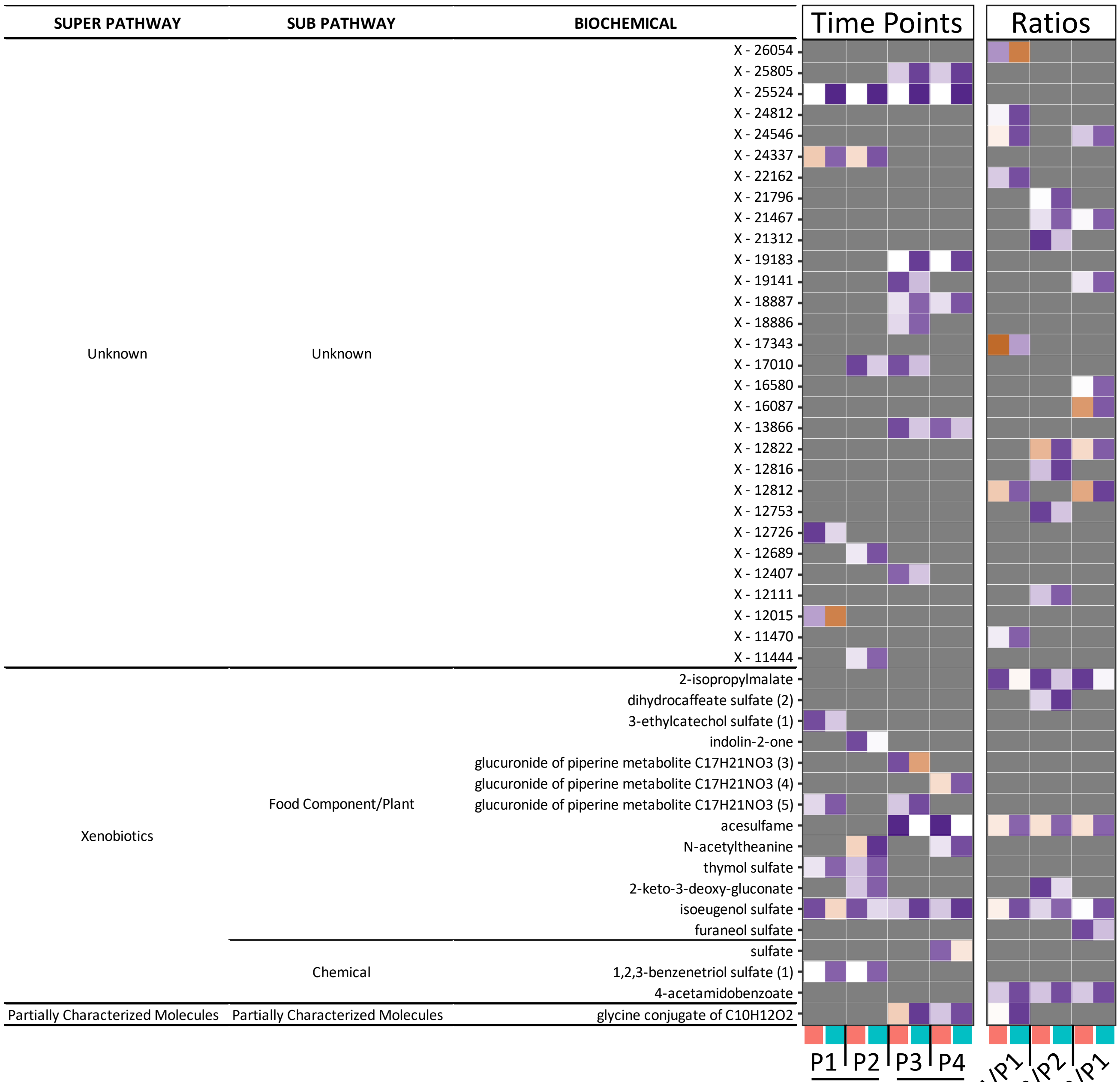

Study Time Point	Urine	Plasma
Baseline	U1	P1
15 min post-CPET1		P2
24 h post-CPET1	U3	P3
15 min post-CPET2		P4

Supplementary Figure 5 – Unknown, partially characterized molecules, and food component / plant compounds with correlations between urine and plasma that are different in ME/CFS patients and controls. The heatmap shows the correlation coefficient (Pearson's R) in both ME/CFS and control groups for compounds and comparisons which meet the following criteria: 1) $|R| > 0.7$, $p < 0.05$, and $q < 0.15$ in either ME/CFS patients or controls; 2) $R < 0.3$ with the same sign or an R value with an opposite sign (i.e. negative if the significant correlation was positive) in the other cohort (controls or patients); 3) compounds that had extreme outliers were removed (modified z-score method of outlier detection, with a threshold of $z > 6$). Within each compound, the R values for the comparisons that did not meet the criteria are not shown (gray boxes). The left panel of the heatmap shows the time point comparisons and the right panel shows the ratio comparisons. The table shows the biochemical name and to which Metabolon subpathway and superpathway it belongs.
